# GALNT10 Affects O‐Glycosylation of IGFBP7 to Promote Tumor Vascular Remodeling and Metastasis of Ovarian Cancer

**DOI:** 10.1002/advs.202516106

**Published:** 2026-02-04

**Authors:** Yanan Zhang, Ayala Zuha, Zhangxin Wu, Aiping Luo, Bixia Jin, Qinkun Sun, Yuan Li, Qiyu Liu, Hongyan Guo, Chunliang Shang

**Affiliations:** ^1^ Department of Obstetrics and Gynecology Peking University Third Hospital Beijing China; ^2^ National Clinical Research Center for Obstetrics and Gynecology Beijing China; ^3^ State Key Laboratory of Vascular Homeostasis and Remodeling Peking University Third Hospital Beijing China; ^4^ State Key Lab of Molecular Oncology National Cancer Center/National Clinical Research Center for Cancer/Cancer Hospital Chinese Academy of Medical Sciences and Peking Union Medical College Beijing China

**Keywords:** GALNT10, O‐GalNAc glycosylation, ovarian cancer, tumor metastasis, tumor vascular remodeling

## Abstract

Tumor metastasis represents a major determinant of prognosis in ovarian cancer. Accumulating evidence has demonstrated that the glycosylation of secretome proteins regulates cell communication in the tumor microenvironment, thereby affecting tumor metastasis; however, the underlying regulatory mechanisms remain unclear. In this study, we observed markedly elevated glycosylation levels in metastatic ovarian cancer and identified GALNT10 as a key glycosyltransferase that promotes EMT of ovarian cancer cells. Furthermore, GALNT10 enhances the extracellular secretion of IGFBP7 through O‐GalNAc glycosylation modification at the T188 site. IGFBP7 subsequently interacts with the CD93 receptor on endothelial cells, leading to vascular remodeling characterized by abnormal vascular formation and impaired vascular maturity. Moreover, we identified the GALNT10 inhibitor Luteolin, which effectively suppresses ovarian cancer metastasis, modulates the immunosuppressive tumor microenvironment through tumor vascular‐immune crosstalk, and exhibits synergistic effects with anti‐PD1 therapy. Collectively, our findings indicate that GALNT10 facilitates ovarian cancer metastasis through the induction of tumor cell EMT and tumor vascular dysfunction, suggesting that GALNT10 inhibitors represent a promising avenue for the development of novel therapeutic strategies in ovarian cancer.

## Graphical abstract

The glycosyltransferase GALNT10 facilitates ovarian cancer metastasis through the induction of tumor cell EMT and tumor vascular dysfunction. GALNT10 enhanced the extracellular secretion of IGFBP7 through O‐GalNAc glycosylation modification at the T188 site, which subsequently interacts with CD93 on endothelial cells, leading to vascular remodeling. The GALNT10 inhibitor Luteolin suppresses ovarian cancer metastasis, modulates the tumor microenvironment through vascular‐immune crosstalk, and exhibits synergistic effects with anti‐PD1 therapy.

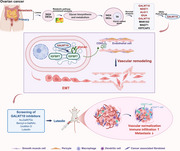

## 1 Introduction

Ovarian cancer is a highly aggressive malignancy and the most lethal gynecological tumor, with a 5‐year survival rate of only 30% [1, 2]. Most patients are diagnosed at an advanced stage, commonly with extensive pelvic and peritoneal metastasis [3]. Tumor metastasis is a critical factor affecting the survival of ovarian cancer patients, presenting significant clinical challenges.

Metabolic reprogramming plays a crucial role in tumor metastasis by modulating the epithelial‐mesenchymal transition (EMT), migration, invasion, anti‐necroptosis, and distant colonization of tumor cells [4, 5]. Furthermore, the metastasis process involves complex interactions between tumor cells and various components of the microenvironment, including cancer‐associated fibroblasts (CAFs), immune cells, endothelial cells, and extracellular matrix (ECM) components [6, 7]. However, the mechanisms by which abnormal metabolism affects tumor cells, modulates the interaction between tumor cells and the tumor microenvironment, and subsequently influences metastasis in ovarian cancer remain to be fully elucidated.

To delineate metabolic mechanisms of ovarian cancer metastasis, we applied Gene Set Variation Analysis (GSVA) to transcriptomic profiles from primary and metastatic ovarian tumors. Our analysis revealed that protein glycosylation represents the predominant metabolic pathway contributing to tumor metastasis. Protein glycosylation refers to the process where glycosyltransferases catalyze the covalent attachment of glycans to amino acid residues of proteins, primarily occurring within the endoplasmic reticulum and Golgi apparatus [8]. Depending on the type of glycan and its linkage to amino acids, glycosylation can be mainly classified into two major types: N‐glycosylation and O‐glycosylation [9]. Aberrant glycosylation is commonly observed in cancer [10], characterized by increased branching of N‐glycans and the expression of truncated O‐glycans. Glycosylation broadly regulates protein subcellular localization, biological activity, and protein‐protein interactions, thereby enhancing tumor cell migration and adhesion and promoting tumor metastasis and invasion [11]. This provides a novel perspective for understanding the complex mechanisms underlying ovarian cancer metastasis. The aberrant glycosylation largely depends on alterations in the expression levels of glycosyltransferases and glycosidases [12]. Therefore, this study aims to identify the key glycosyltransferase and elucidate the underlying mechanism driving ovarian cancer metastasis.

Our study demonstrated that glycosyltransferase GALNT10 plays a critical role in the metastasis of ovarian cancer. As a member of the GALNT family, GALNT10 catalyzes the initiating step of O‐GalNAc glycosylation [13, 14]. Previous studies have investigated the roles of GALNT family members in tumorigenesis and demonstrated their critical involvement in tumor metastasis. For example, GALNT6‐mediated aberrant O‐glycosylation of prohibitin 2 promotes clear cell renal cell carcinoma progression [15]. Aberrant O‐glycosylation mediated by GALNT6 has also been shown to promote EMT in tumor cells and to reduce CD8+ T cell infiltration [16]. GALNT9‐mediated O‐GalNAc glycosylation of Annexin A2 activates the mannose‐binding lectin complement pathway in the liver, promoting hepatic metastasis of neuroendocrine prostate cancer [17]. GALNT14 enhances the initiation and growth of lung metastatic colonies, thereby driving breast‐to‐lung dissemination [18]. However, the function and mechanism by which GALNT10 promotes tumor metastasis remain poorly understood.

We found that high GALNT10 expression levels in tumors are associated with poor prognosis in ovarian cancer patients. Further investigation revealed that GALNT10‐mediated glycosylation modification of IGFBP7 enhances its extracellular secretion. Previous studies have shown that IGFBP7 interacts with CD93 as a ligand‐receptor pair [19]. In this study, we uncovered that secreted IGFBP7 interacts with CD93 on the surface of vascular endothelial cells, inducing abnormal angiogenesis and insufficient vascular maturation, which collectively promote ovarian cancer metastasis. Additionally, we identified Luteolin as a GALNT10 inhibitor. Both in vitro and in vivo experiments demonstrated that Luteolin promotes vascular normalization, enhances immune infiltration through the tumor vascular‐immune crosstalk, and effectively suppresses ovarian cancer metastasis.

## 2 Results

### 2.1 Glycosylation is the Predominantly Enriched Metabolic Pathway Associated with Ovarian Cancer Metastasis, and the Glycotransferase GALNT10 Plays a Critical Role

**FIGURE 1:**
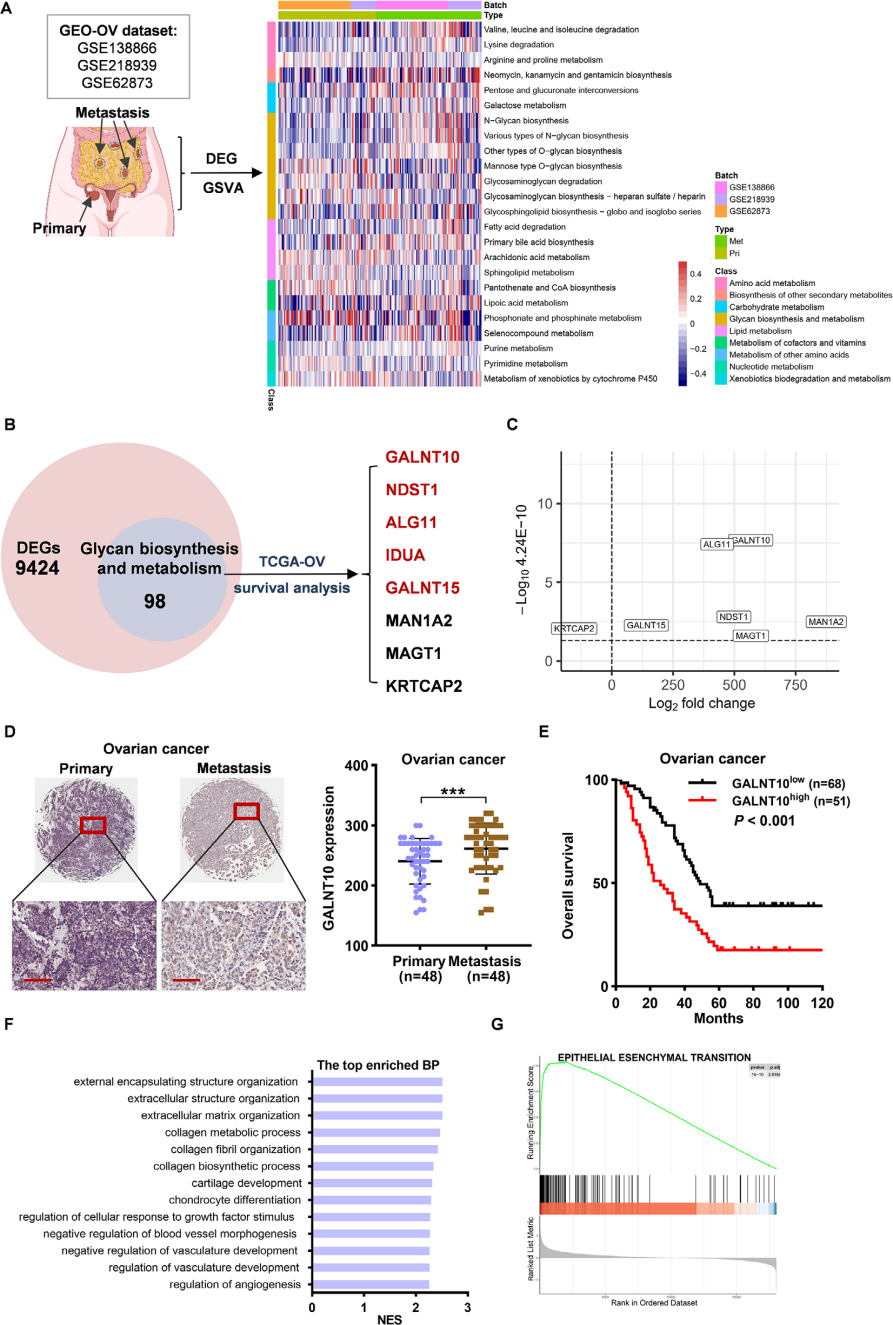
The glycosyltransferase GALNT10 is identified as a key regulator associated with ovarian cancer metastasis. (A) Metabolism‐related pathways enrichment of DEGs between metastatic and primary tumors in the GEO‐OV datasets based on the GSVA scores via the Wilcoxon rank‐sum test. (B) Among DEGs of metastatic and primary ovarian cancer, 98 genes related to glycan biosynthesis and metabolism were identified, with 8 showing a significant association with OS in the TCGA‐OV dataset based on Kaplan‐Meier analysis. (C) The Log2 fold change values of 8 glycan‐associated genes in the metastatic tumor and the primary tumor in the GEO‐OV datasets. (D) The GALNT10 expression in primary and metastatic ovarian cancer. The scale shown in the picture is 100 µm. Statistical analysis was performed by a two‐tailed, unpaired Student's t‐test. ^***^ *p* < 0.001. (E) Survival analysis of GALNT10 expression in ovarian cancer tissue microarray samples using the Kaplan‐Meier method. (F,G) BP and HALLMARK pathway enrichment of DEGs between GALNT10^high^ and GALNT10^low^ groups in the TCGA‐OV dataset.

Ovarian cancer is marked by extensive metabolic reprogramming [20]. To investigate the key metabolic alterations associated with ovarian cancer metastasis, we first analyzed differentially expressed genes (DEGs) between metastatic and primary tumors using the Gene Expression Omnibus (GEO) datasets GSE218939, GSE62873, and GSE138866. Specifically, GSE218939 includes 39 paired samples of primary and metastatic tumors, GSE62873 comprises 63 primary tumor cases, and GSE138866 contains 130 metastatic tumor samples. We identified 9424 DEGs between the metastatic and primary groups, which were enriched in 24 metabolic pathways. Among these, glycosylation‐related pathways were predominantly enriched, including N‐glycan biosynthesis, O‐glycan biosynthesis, and glycosphingolipid biosynthesis (Figure 1A). Given that alterations in glycosyltransferases and glycosidases predominantly affect glycosylation patterns [12], we focused on genes encoding glycosyltransferases. Further analysis revealed that 98 genes related to glycan biosynthesis and metabolism exhibited significantly altered expression levels between metastatic and primary tumors. Survival analysis of the TCGA ovarian cancer dataset demonstrated that GALNT10, NDST1, ALG11, IDUA, GALNT15, MAN1A2, MAGT1, and KRTCAP2 were significantly associated with overall survival (OS) (Figure 1B). Notably, GALNT10, NDST1, ALG11, IDUA, and GALNT15 were identified as poor prognostic factors (Figure S1A–G). Additionally, in the GEO dataset, GALNT10 was identified as the most significantly upregulated glycosyltransferase in metastatic tumors (Figure 1C). To verify the clinical significance of GALNT10 in ovarian cancer, we assessed its expression in ovarian cancer tissue microarrays by immunohistochemistry. In a tissue microarray comprising 48 paired samples of primary and metastatic lesions, GALNT10 expression was significantly higher in metastatic tumors compared to primary tumors (Figure 1D). In another tissue microarray containing primary tumors from 119 ovarian cancer patients, the GALNT10^high^ group showed significantly shorter OS than the GALNT10^low^ group (Figure 1E). The multivariate Cox regression analysis demonstrated that GALNT10 expression serves as an independent prognostic factor for OS (Figure S2). Biological process (BP) and HALLMARK functional enrichment analysis revealed that tumor metastasis‐associated pathways such as extracellular structure organization, extracellular matrix organization, angiogenesis, and EMT were significantly enriched in the GALNT10^high^ group (Figure 1F,G). Collectively, these findings suggest that GALNT10 is a critical glycosyltransferase influencing ovarian cancer metastasis and survival.

### 2.2 GALNT10 Facilitates Ovarian Cancer Metastasis Through the Induction of EMT and Enhancement of Tumor Cell Motility

**FIGURE 2:**
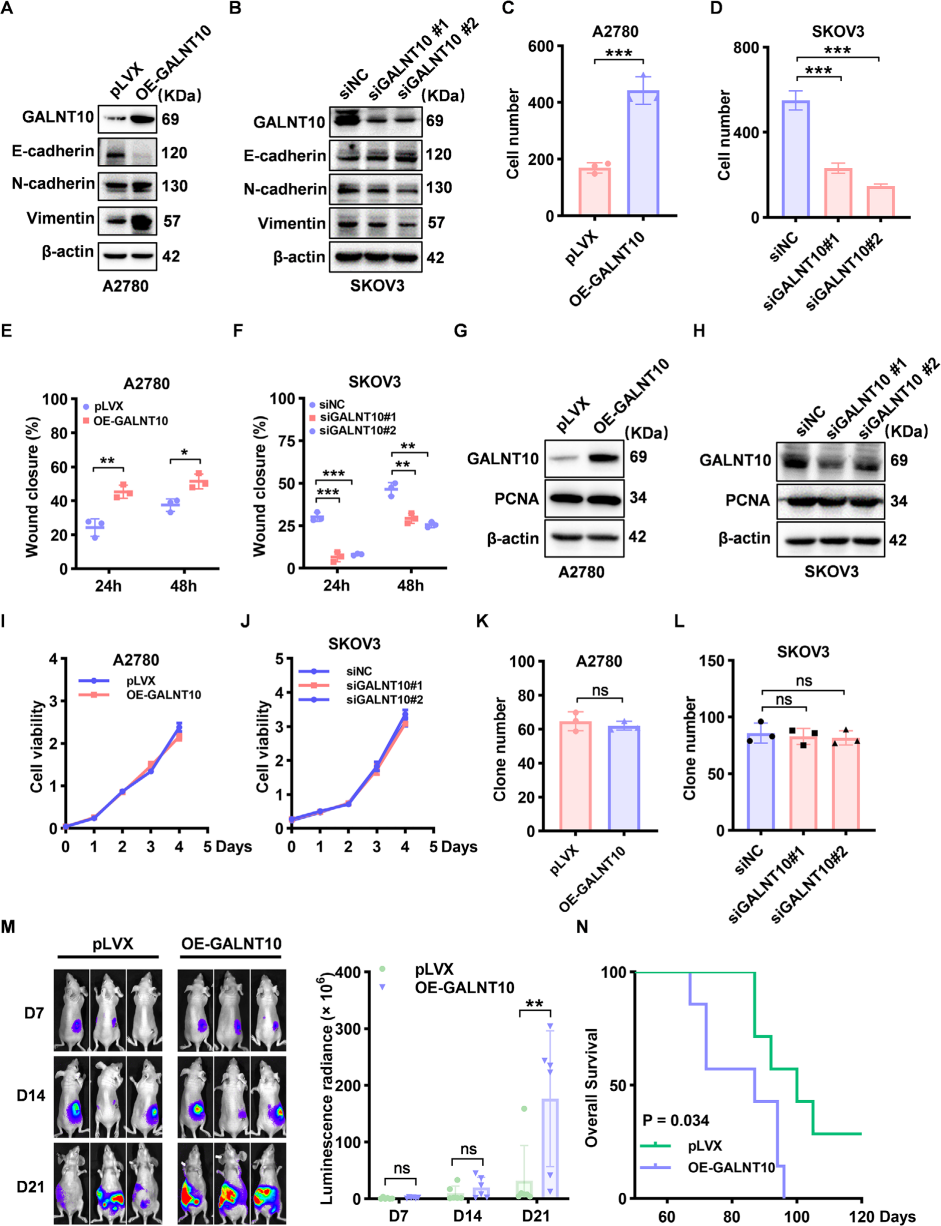
GALNT10 promotes ovarian cancer metastasis in vitro and in vivo. (A) GALNT10 and EMT markers expression in A2780 cells transiently transfected with GALNT10 plasmid or control pLVX plasmid was detected by Western blot. (B) GALNT10 and EMT markers expression in SKOV3 cells transiently transfected with GALNT10 siRNA (siGALNT10 #1‐2) or negative control siRNA (siNC) was detected by Western blot. (C,E) The invasion and migration abilities of A2780 cells transiently transfected with GALNT10 plasmid or control pLVX plasmid were analyzed by Transwell (C) and wound healing (E). (D,F) The invasion and migration abilities of SKOV3 cells transiently transfected with siGALNT10 #1‐2 or negative control siNC were analyzed by Transwell (D) and wound healing (F). (G) PCNA in A2780 cells transiently transfected with GALNT10 plasmid or control pLVX plasmid was detected by Western blot. (H) PCNA in SKOV3 cells transiently transfected with siGALNT10 #1‐2 or negative control siNC was detected by Western blot. (I,K) Cell proliferation of A2780 cells transiently transfected with GALNT10 plasmid or control pLVX plasmid was detected by CCK8 (I) and clone formation (K). (J,L) Cell proliferation of SKOV3 cells transiently transfected with siGALNT10 #1‐2 or negative control siNC was detected by CCK8 (J) and clone formation (L). (M) Representative bioluminescence images of mice bearing orthotopic A2780‐luc cells with GALNT10 overexpression or with control tumors, and the quantification of the bioluminescence intensities (n = 6 mice per group). (N) Survival of mice bearing orthotopic A2780 cells with GALNT10 overexpression or with control tumors. Statistical analysis for figure M was conducted using the Mann‐Whitney U test, while all other analyses were performed using the two‐tailed, unpaired Student's t‐test. ^**^ *p* < 0.01, ^***^ *p* < 0.001, ns represents P > 0.05.

**FIGURE 3:**
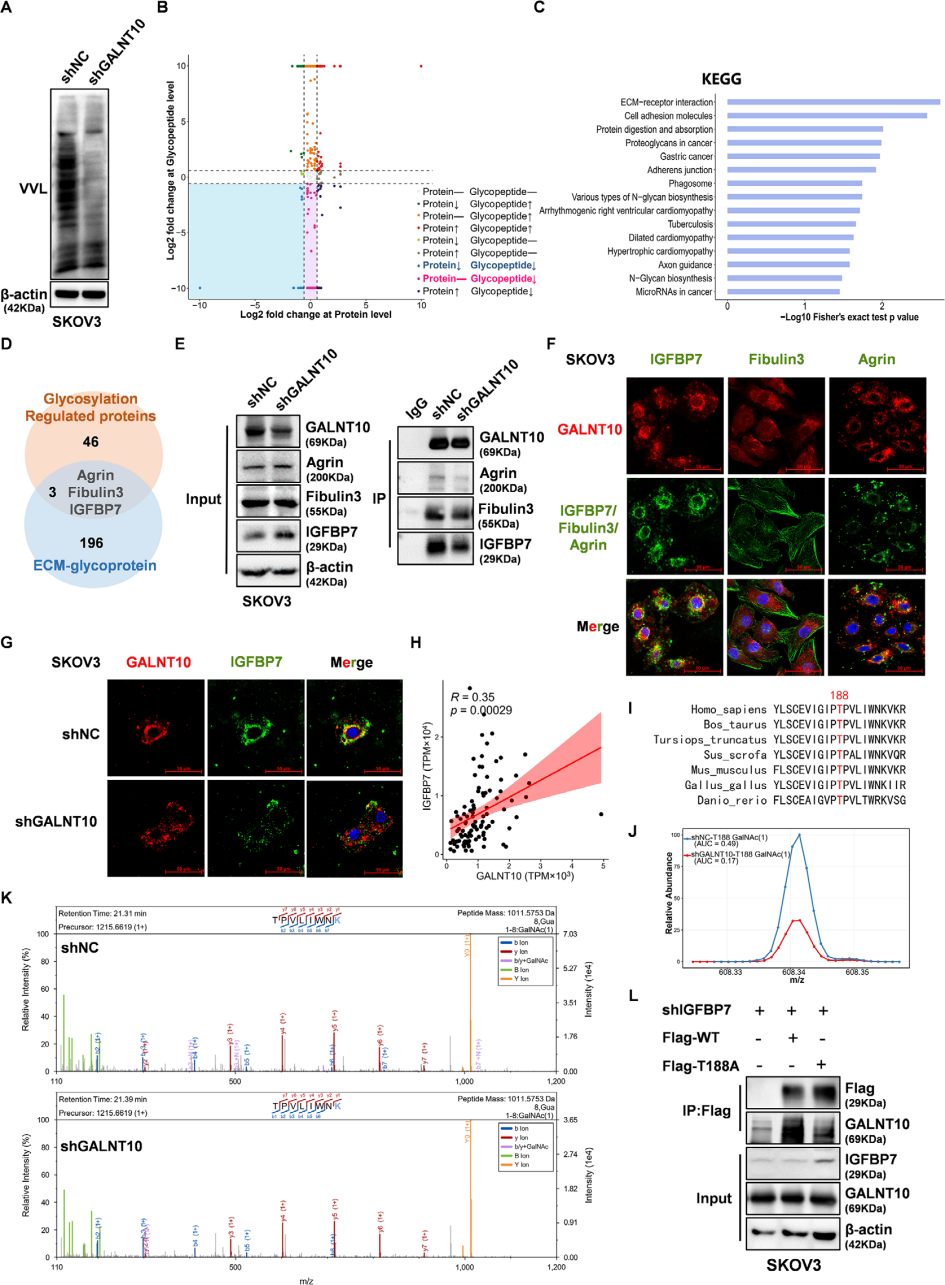
GALNT10 regulates the glycosylation modification of IGFBP7. (A) O‐GalNAc glycosylation level of SKOV3 cells with GALNT10 knockdown by shRNA (shGALNT10) or negative control (shNC) was detected by VVL blot. (B) The O‐GalNAc glycopeptides changes between shGALNT10 SKOV3 cells and shNC SKOV3 cells were detected via O‐GalNAc glycoproteomics. (C) KEGG pathway enrichment of 46 proteins with decreased O‐GalNAc glycosylation levels in shGALNT10 SKOV3 cells. (D) 3 ECM‐related glycoproteins among the 46 O‐GalNAc glycosylation changed proteins. (E) The interaction between GALNT10 and Agrin, Fibulin3, and IGFBP7 in shGALNT10 SKOV3 cells and shNC SKOV3 cells was analyzed with Co‐IP and Western blot. (F) The co‐localization between GALNT10 and Agrin, Fibulin3, and IGFBP7 was detected by immunofluorescence. (G) The co‐localization between GALNT10 and IGFBP7 in shGALNT10 SKOV3 cells and shNC SKOV3 cells was detected by immunofluorescence. The scale shown in the picture of F and G is 50 µm. (H) Correlation analysis of GALNT10 with IGFBP7 based on RNA‐seq data of 102 ovarian cancer tissues. (I) The amino acid sequences of the IGFBP7 protein containing the predicted glycosylation site (T188, in red) across different species. (J) The primary quantitative extracted ion chromatograms (XICs) of the GalNAc(1) glycopeptide at the T188 site of IGFBP7 in SKOV3 shNC cells and shGALNT10 cells. (K) The GalNAc(1) glycan structures on glycoprotein IGFBP7 at T188 in SKOV3 shNC cells and shGALNT10 cells were characterized by glycoproteomics. (L) Interaction between GALNT10 and IGFBP7 in WT‐IGFBP7 or T188A‐IGFBP7 SKOV3 cells was analyzed with Co‐IP and Western blot.

To confirm the role of GALNT10 in ovarian cancer metastasis, we conducted a series of in vitro and in vivo experiments. First, we examined GALNT10 expression levels in ovarian cancer cell lines using Western blot and found that SKOV3 cells exhibited high GALNT10 expression, whereas A2780 cells showed low expression (Figure S3). Subsequently, we overexpressed GALNT10 with a plasmid and knocked down GALNT10 using specific siRNAs. The Western blot and the Transwell assays revealed that GALNT10 overexpression in A2780 cells significantly upregulated EMT markers and enhanced cell invasion and migration abilities (Figure 2A,C,E; Figure S4A,B), while GALNT10 knockdown in SKOV3 cells markedly inhibited EMT and cell invasion and migration abilities (Figure 2B,D,F; Figure S4C,D). Additionally, we evaluated the effect of GALNT10 on cell proliferation by detecting the expression of the proliferation marker PCNA via Western blot and performing CCK8 and colony formation assays. All above demonstrated that GALNT10 had no significant impact on cell proliferation, confirming that its promotion of tumor metastasis was not attributable to increased proliferation (Figure 3G–L; Figure S5A,B). To further validate these findings in vivo, we injected luciferase‐labeled A2780 cells with or without GALNT10 overexpression into the ovarian bursa of mice to establish the orthotopic ovarian cancer model and monitored tumor metastasis using bioluminescent imaging. We observed that the GALNT10‐overexpression group exhibited more extensive metastasis and poorer survival compared to the control group (Figure 2M,N). In conclusion, GALNT10 may serve as a therapeutic target for ovarian cancer metastasis.

### 2.3 GALNT10 regulates O‐GalNAc Glycosylation of IGFBP7 at the T188 Site

Next, we investigated the mechanism of GALNT10 promoting ovarian cancer metastasis. First, we knocked down GALNT10 in SKOV3 cells using shRNA. VVL Lectin Blot analysis revealed that the O‐GalNAc glycosylation level was decreased in shGALNT10 SKOV3 cells (Figure 3A). Subsequently, we performed an O‐GalNAc glycoproteomic analysis to identify glycopeptides affected by GALNT10 knockdown. This analysis identified 46 proteins that exhibited reduced O‐GalNAc glycosylation (with either unchanged or reduced protein levels) in shGALNT10 SKOV3 cells compared to shNC SKOV3 cells (Figure 3B, Table S1). Kyoto Encyclopedia of Genes and Genomes (KEGG) and Gene Ontology (GO) functional enrichment analyses indicated that these proteins were enriched in pathways such as ECM‐receptor interaction, proteoglycans in cancer, and tumor cell migration (Figure 3C, Figure S6A). The ECM‐related genes annotated in the Gene Set Enrichment Analysis (GSEA) database include a total of 196 glycoproteins (Table S2). Among the 46 identified proteins, Agrin, Fibulin3, and IGFBP7 were ECM‐related glycoproteins (Figure 3D). Co‐IP experiments confirmed that GALNT10 strongly interacted with Fibulin3 and IGFBP7, with the interaction between GALNT10 and IGFBP7 significantly reduced in shGALNT10 SKOV3 cells (Figure 3E). Further immunofluorescence experiments validated that IGFBP7 co‐localizes with GALNT10, whereas the co‐localization between GALNT10 and Agrin or Fibulin3 was weak (Figure 3F). The immunofluorescence experiments showed that the co‐localization of GALNT10 and IGFBP7 was decreased in the shGALNT10 SKOV3 cells compared with shNC SKOV3 cells (Figure 3G). Molecular docking analysis showed that GALNT10 and IGFBP7 exhibited strong binding activity, with a binding energy of ‐7.1 kcal/mol (Figure S6B). And the IGFBP7 expression was positively correlated with GALNT10 both in our cohort of 102 ovarian cancer tissues and in the TCGA ovarian cancer dataset (Figure 3H; Figure S6C). Glycoproteomics analysis revealed differential O‐GalNAc glycosylation at the T188 site of IGFBP7, a conserved and putative O‐glycosylation site across species (Figure 3I). Two GalNAc‐modified glycoforms were identified at the T188 site: GalNAc(1) and GalNAc(1)Hex(1). Compared with the shNC group, the abundance of the GalNAc(1) glycoform was significantly reduced in the shGALNT10 group (Figure 3J,K), and the GalNAc(1)Gal(1) glycoform was even undetectable in the shGALNT10 group (Figure S6D,E). We then established SKOV3 cells with the IGFBP7 T188 mutation to confirm the O‐GalNAc glycosylation site. Firstly, endogenous IGFBP7 was stably knocked down by shRNA lentiviral transduction (Figure S6F), and shIGFBP7#2 SKOV3 cells with the highest efficiency were selected for further experiments. Secondly, wild‐type (WT) or the T188A mutant (Thr188 to Ala188) IGFBP7 plasmids with Flag‐tag were transiently overexpressed in shIGFBP7#2 SKOV3 cells to generate WT or T188A mutant cells, respectively. Subsequent Co‐IP experiments demonstrated that the binding of IGFBP7 to GALNT10 was significantly weakened in T188A cells (Figure 3L). These findings demonstrate that O‐GalNAc glycosylation of IGFBP7 at the T188 residue is regulated by GALNT10.

### 2.4 GALNT10‐Mediated IGFBP7 Glycosylation Promotes Tumor Cell Motility and Vascular Remodeling in Ovarian Cancer

**FIGURE 4:**
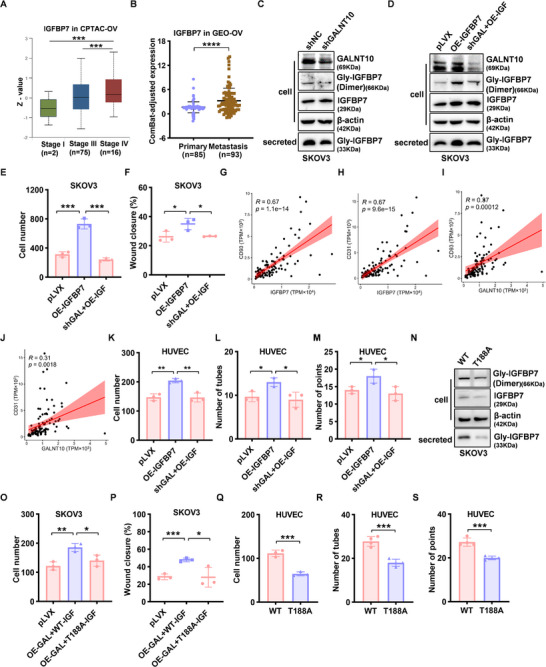
IGFBP7 glycosylated by GALNT10 promotes ovarian cancer metastasis through increased tumor cell migration and angiogenesis. (A) Correlation of IGFBP7 level and stage in CPTAC‐OV dataset. (B) The correlation between IGFBP7 level and metastasis in the GEO‐OV dataset. (C) The glycosylated and secreted IGFBP7 protein in shGALNT10 SKOV3 cells and control shNC SKOV3 cells was analyzed with Western blot. (D) The glycosylated and secreted IGFBP7 protein in OE‐IGFBP7 SKOV3 cells, shGALNT10+OE‐IGFBP7 SKOV3 cells, and control SKOV3 cells were analyzed with a Western blot. (E, F) The invasion and migration abilities of OE‐IGFBP7 SKOV3 cells, shGALNT10+OE‐IGFBP7 SKOV3 cells, and control SKOV3 cells were analyzed by Transwell (E) and wound healing (F). (G,H) Correlation analysis of IGFBP7 with CD93 and CD31 based on RNA‐seq data of 102 ovarian cancer tissues. (I,J) Correlation analysis of GALNT10 with CD93 and CD31 based on RNA‐seq data of 102 ovarian cancer tissues. (K) The invasion abilities of HUVECs incubated with CM of OE‐IGFBP7 SKOV3 cells, shGALNT10+OE‐IGFBP7 SKOV3 cells, and control SKOV3 cells for 24 h were analyzed by Transwell. (L,M) The angiogenesis ability of HUVECs incubated with CM of OE‐IGFBP7 SKOV3 cells, shGALNT10+OE‐IGFBP7 SKOV3 cells, and control SKOV3 cells for 24 h was analyzed by Tube formation. (N) The glycosylated and secreted IGFBP7 level of SKOV3 shIGFBP7 cells transiently transfected with WT or the T188A mutant IGFBP7 plasmids was detected by Western blot. (O,P) The invasion and migration abilities of SKOV3 shIGFBP7 cells transiently transfected with GALNT10 plasmid, WT‐IGFBP7 or T188A‐IGFBP7, or control pLVX plasmid were analyzed by Transwell (O) and wound healing (P). (Q) The invasion abilities of HUVECs incubated with CM of WT‐IGFBP7 SKOV3 cells and T188A‐IGFBP7 SKOV3 cells for 24 h were analyzed by Transwell. (R, S) The angiogenesis ability of HUVECs incubated with CM of WT‐IGFBP7 SKOV3 cells and T188A‐IGFBP7 SKOV3 cells for 24 h was analyzed by Tube formation. Statistical analysis was performed by a two‐tailed, unpaired Student's t‐test. ^*^ *p* < 0.05, ^**^ *p* < 0.01, ^***^ *p* < 0.001, ^****^ *p* < 0.0001.

The effects of glycosylation on IGFBP7 function and ovarian cancer metastasis have never been explored. IGFBP7 is a member of the IGF‐binding protein (IGFBP) family, a group of secreted proteins that mainly bind to insulin and insulin‐like growth factors (IGFs) through the N‐terminal IB domain, regulating the binding of IGF to its receptors, and thereby influencing cell proliferation, apoptosis, adhesion, etc [21]. Proteomic data from the Clinical Proteomic Tumor Analysis Consortium (CPTAC) database demonstrated that IGFBP7 is significantly upregulated in multiple tumors compared to normal tissues (Figure S7A), with a notably increased level observed in advanced‐stage ovarian cancer (Figure 4A). Analysis of GEO datasets (GSE218939, GSE62873, and GSE138866) revealed that IGFBP7 expression is markedly higher in metastatic tumors than in primary tumors (Figure 4B). The survival analysis of the combined TCGA and GEO ovarian cancer dataset shows that the IGFBP7^high^ group exhibited significantly shorter OS than the IGFBP7^low^ group (Figure S7B). Collectively, these findings indicate that IGFBP7 is associated with tumor metastasis and survival.

To further explore the effect of glycosylated IGFBP7, we collected cellular and supernatant proteins from GALNT10 knockdown (shGALNT10) and negative control (shNC) SKOV3 cells. Western blot revealed that glycosylated IGFBP7 and secreted IGFBP7 in the supernatant were both reduced in the shGALNT10 group (Figure 4C). The molecular weight of IGFBP7 secreted into the extracellular space was determined to be 33 kDa, consistent with its glycosylated form, which is higher than that of the non‐glycosylated counterpart (29 kDa). Furthermore, intracellular glycosylated IGFBP7 predominantly exists as a dimer, a characteristic that aligns well with previous reports [22]. Next, we constructed SKOV3 cells with IGFBP7 overexpression (OE‐IGFBP7), with IGFBP7 overexpression and GALNT10 knockdown (shGALNT10+OE‐IGFBP7). Western Blot showed that glycosylated and secreted IGFBP7 was also increased in the IGFBP7 overexpression cells, while GALNT10 knockdown reduced glycosylated IGFBP7 and secreted IGFBP7 (Figure 4D). The Transwell and wound healing assays demonstrated that IGFBP7 overexpression significantly enhanced the cell invasion and migration capabilities, which could be reversed by GALNT10 knockdown (Figure 4E,F; Figure S7C,D).

A recent study confirmed that IGFBP7 induces vascular homeostasis imbalance and promotes tumor progression by binding to CD93 on endothelial cells [19, 23]. Protein‐protein interaction (PPI) analysis indicated that IGFBP7 interacts with CD93 (Figure S8A), and molecular docking analysis further confirmed strong binding activity between IGFBP7 and CD93, with a binding energy of −7.0 kcal/mol (Figure S8B). Correlation analysis of ovarian cancer tissues revealed that the expression levels of both IGFBP7 and GALNT10 were positively correlated with CD93 and CD31, a marker of vascular endothelial cells (Figure 4G–J; Figure S8C–F). To investigate the regulatory effect of IGFBP7 on angiogenesis, the supernatant from SKOV3 cells with OE‐IGFBP7 or with shGALNT10+OE‐IGFBP7 was collected to incubate human umbilical vein endothelial cells (HUVECs). The results revealed that incubation with the supernatant of IGFBP7‐overexpressing cells enhanced migration and tube formation capabilities of HUVECs, which could be reversed by GALNT10 knockdown (Figure 4K–M; Figure S8G,H). The levels of glycosylated and secreted IGFBP7 were reduced in T188A mutant cells compared to wild‐type cells (Figure 4N), which reversed not only the enhanced cell invasion and migration induced by GALNT10 overexpression (Figure 4O,P; Figure S9A,B), but also the migratory and tube‐forming capacities of HUVECs (Figure 4Q–S; Figure S9C,D). Collectively, the aforementioned findings suggest that IGFBP7 may undergo glycosylation at the T188 site mediated by GALNT10, which could be essential for its functional activity in facilitating the metastasis of ovarian cancer by promoting tumor cells' motility and vascular remodeling.

### 2.5 The GALNT10 inhibitor Luteolin Prevents Ovarian Cancer Metastasis

**FIGURE 5:**
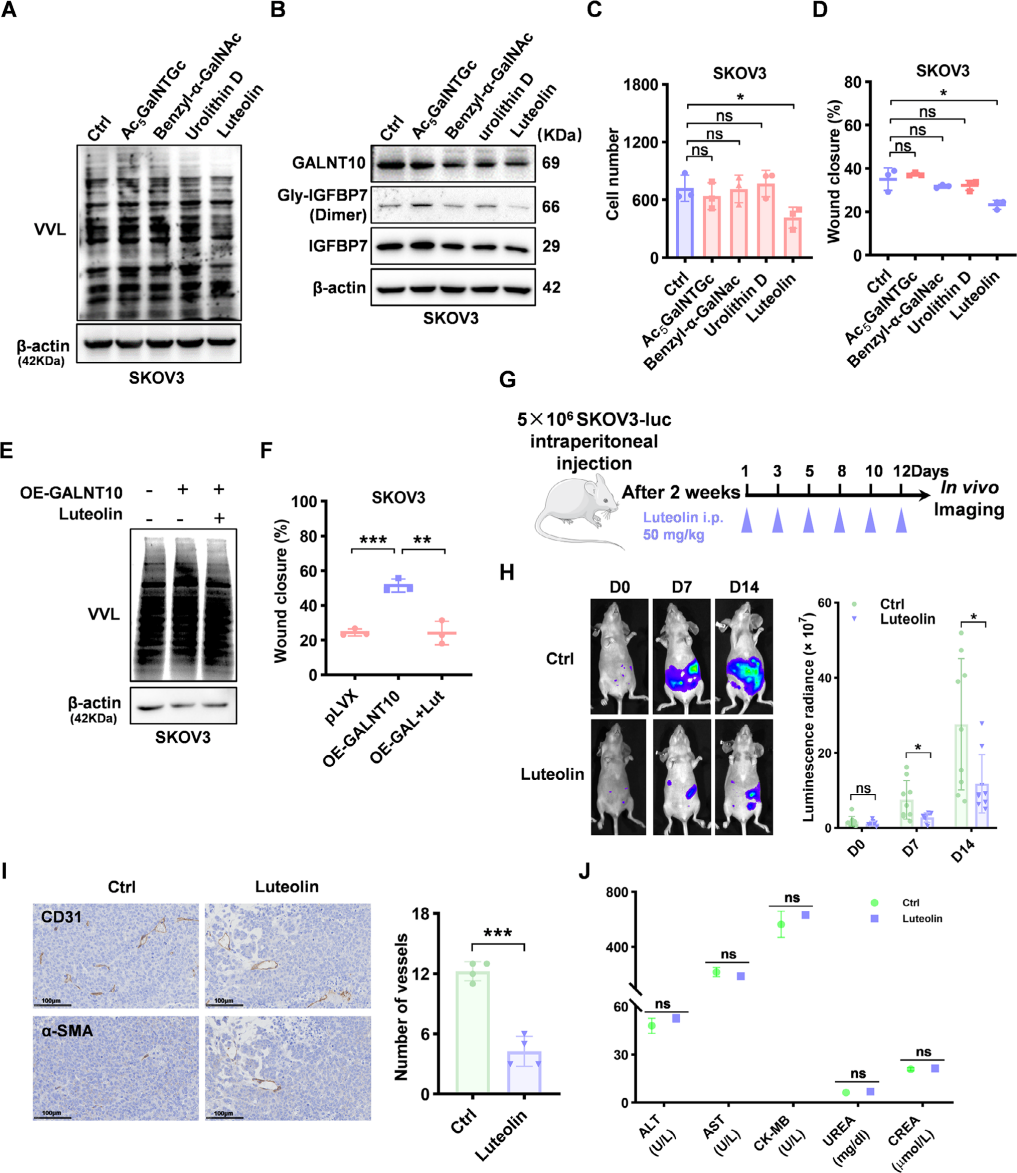
The GALNT10 inhibitor Luteolin prevents ovarian cancer metastasis. (A) The O‐glycosylation inhibition of GALNTs inhibitors in SKOV3 cells was detected by VVL Blot. (B) The glycosylated IGFBP7 level inhibition of GALNTs inhibitors in SKOV3 cells was detected by Western Blot. (C,D) The invasion and migration abilities inhibition of GALNTs inhibitors in SKOV3 cells were analyzed by Transwell (C) and wound healing (D). (E) The O‐glycosylation of GALNT10 overexpressing and Luteolin treatment of SKOV3 cells was detected by VVL Blot. (F) The migration of GALNT10 overexpressing and Luteolin treatment of SKOV3 cells was detected by wound healing. (G) Schematic illustration of therapeutic experiments in peritoneal metastasis ovarian cancer models. (H) Representative bioluminescence images of mice bearing abdominal SKOV3‐derived tumors after treatment as indicated and the quantification of the bioluminescence intensities (n = 9 mice per group). (I) CD31 and α‐SMA expression of tumors was detected by IHC. The quantification of vessels according to the CD31 and α‐SMA expression (n = 4). The scale shown in the picture is 100 µm. (J) Representative blood biochemistry tests were carried out in the mice. The quantification is shown as the mean ± SD (n = 3 mice per group). Statistical analysis for Figure H was conducted using the Mann‐Whitney U test, while all other analyses were performed using the two‐tailed, unpaired Student's t‐test. ^*^ *p* < 0.05, ^**^ *p* < 0.01, ^***^ *p* < 0.001, ns represents P > 0.05.

After identifying GALNT10 as a key glycosyltransferase promoting ovarian cancer metastasis, we further investigated the anti‐metastatic effects of GALNT10 inhibitors. We conducted an in vitro screening of previously identified inhibitors targeting the GALNTs family, including Luteolin, Acetyl N‐thioethyl‐D‐galactosamine (Ac_5_GalNTGc), Aryl‐α‐N‐acetylgalactosamine (Benzyl‐α‐GalNAc), and Urolithin D [24]. First, the IC50 values of Ac5GalNTGc, Benzyl‐α‐GalNAc, Luteolin, and Urolithin D were determined using the CCK8 assay, and all were found to exceed 20 µm (Figure S10A). When SKOV3 cells were treated with these inhibitors at a concentration of 10 µm, the VVL Lectin Blot experiment revealed that Luteolin exhibited the most significant O‐glycosylation inhibition of SKOV3 cells (Figure 5A). Western Blot showed that Luteolin significantly reduced glycosylation of IGFBP7 (Figure 5B; Figure S10B). The Transwell and wound healing assays confirmed that Luteolin exerted the strongest inhibition on cell migration and invasion (Figure 5C,D; Figure S10C,D). Liu et al. have evaluated the effect of Luteolin on the GALNT family, and the glycosyltransferase activity experiments showed that Luteolin showed the most potent inhibition on GALNT10 with an IC50 of 1.7 µm, significantly lower than the IC50 of other family members [25].

We established GALNT10‐overexpressing cells and observed that the upregulated glycosylation (Figure 5E) and the pro‐metastatic effects were inhibited by Luteolin, confirming its specific inhibitory effect on GALNT10(Figure 5F; Figure S10E). In the peritoneal metastatic ovarian cancer model constructed with luciferase‐labeled SKOV3 cells, intraperitoneal administration of 50 mg/kg Luteolin significantly inhibited tumor metastasis after 7 days and 14 days of treatment (Figure 5G,H; Figure S11A). Continuous sectioning of tumor tissue and subsequent immunohistochemical staining for CD31 and α‐SMA revealed an abnormal tumor angiogenesis alongside a loss of vascular maturation in the control group. In contrast, Luteolin inhibited angiogenesis and promoted vascular normalization and maturation (Figure 5I). Additionally, blood biochemical indexes, mice weight measurement, and H&E staining images of the major organs showed that the Luteolin therapy was safe (Figure 5J; Figure S11B, C). All of the above results collectively confirm that Luteolin, as a GALNT10 inhibitor, effectively suppresses ovarian cancer metastasis and exhibits good safety.

### 2.6 The GALNT10 Inhibitor Luteolin Promotes Immune Infiltration and Exerts a Synergistic Effect with Anti‐PD1 Therapy

**FIGURE 6:**
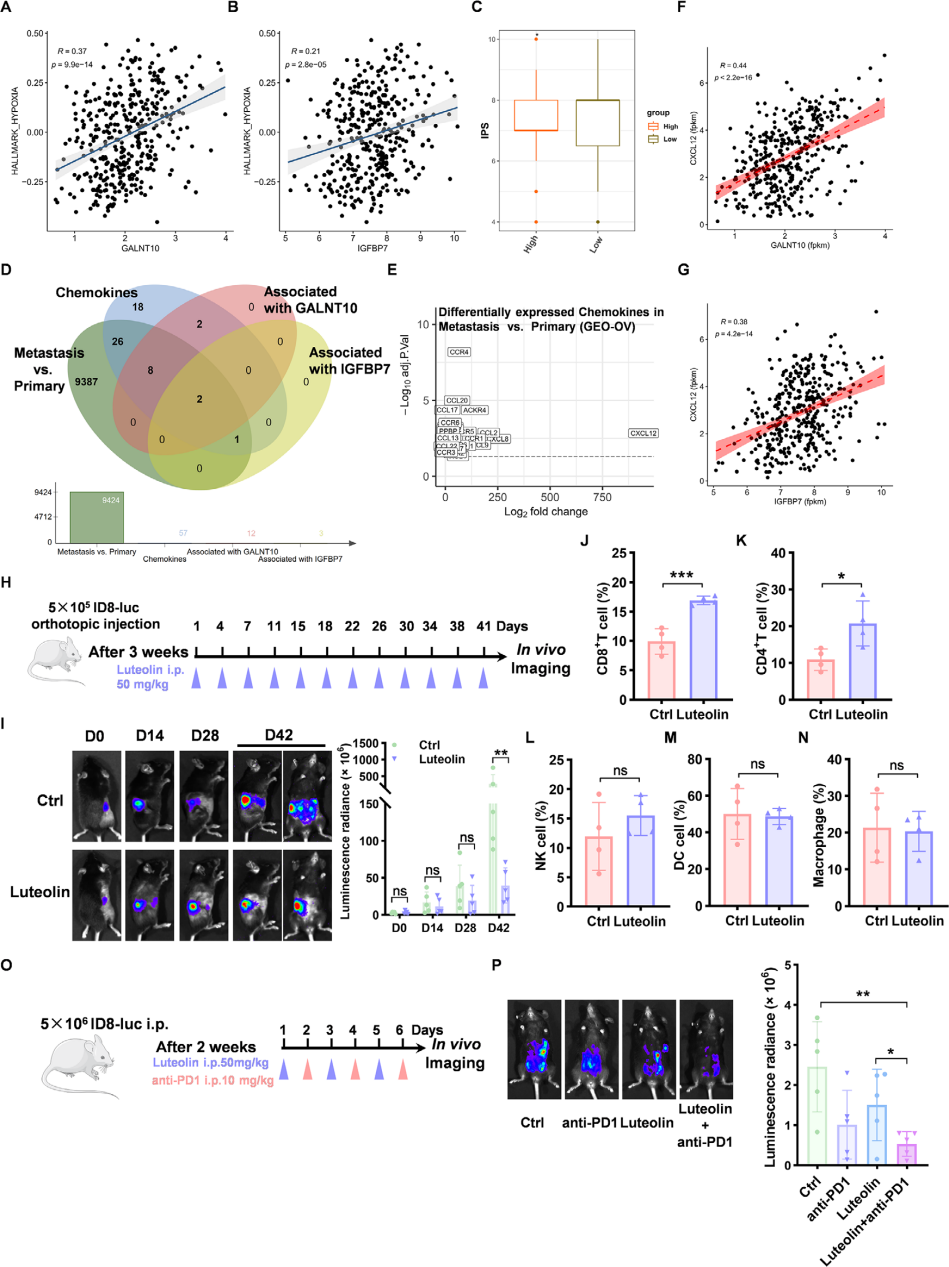
Luteolin promotes immune infiltration and exerts a synergistic effect with anti‐PD1 therapy. (A) Correlation analysis of GALNT10 and hypoxia‐related‐genes in the TCGA‐OV dataset. (B) Correlation analysis of IGFBP7 and hypoxia‐related‐genes in the TCGA‐OV dataset. (C) The IPS score of GALNT10^high^ and GALNT10^low^ groups in the TCGA‐OV dataset. (D) The Venn diagram illustrates the chemokines associated with ovarian cancer metastasis and co‐expressed with both GALNT10 and IGFBP7. (E) The Log2 fold change values of 23 chemokines in the metastatic tumor and the primary tumor in the GEO‐OV datasets. (F, G) Correlation analysis of GALNT10, IGFBP7, and CXCL12 in the TCGA‐OV dataset. (H) Schematic illustration of therapeutic experiments in orthotopic ovarian cancer models. (I) Representative bioluminescence images of mice bearing ID8‐derived orthotopic tumors after treatment as indicated and the quantification of the bioluminescence intensities (n = 5 mice per group). (J–N) The proportions of immune cells in tumors were detected by CyteFlow analysis (n = 4 mice per group). (O) Schematic illustration of therapeutic experiments in peritoneal metastasis ovarian cancer models. (P) Representative bioluminescence images of mice bearing abdominal ID8‐derived tumors after treatment as indicated and the quantification of the bioluminescence intensities (n = 5 mice per group). Statistical analysis for Figure I was conducted using the Mann‐Whitney U test, while all other analyses were performed using the two‐tailed, unpaired Student's t‐test. ^*^ *p* < 0.05, ^**^ *p* < 0.01, ^***^ *p* < 0.001, ns represents P > 0.05.

Abnormal tumor vasculature is regarded as a key contributor to the elevated secretion of hypoxia‐inducing and immunosuppressive factors, leading to the establishment of an immunosuppressive tumor microenvironment. Therefore, we investigate the effect of GALNT10‐IGFBP7‐mediated vascular remodeling on hypoxia and immune conditions. Analysis of the TCGA‐OV dataset reveals a significant correlation between GALNT10 and IGFBP7 expression and hypoxia‐related gene signatures (Figure 6A,B). CIBERSORT analysis of TCGA‐OV samples identified 6 immune cell types with significantly distinct distributions across GALNT10^high^ and GALNT10^low^ groups. Among these, the activation of NK cells was reduced, while M2 macrophage polarization was enhanced in the GALNT10^high^ group (Figure S12A). We observed that the IPS score was lower in the GALNT10^high^ group (Figure 6C), indicating that GALNT10 is linked to an immunosuppressive microenvironment.

Accumulating evidence indicates that hypoxia‐induced alterations in chemokine expression—well‐documented to influence immune cell infiltration patterns—play a pivotal role in shaping antitumor immunity. [26] We identified candidate chemokines associated with ovarian cancer metastasis and the GALNT10‐IGFBP7 axis (Figure 6D). Among a dataset of 57 chemokine‐related genes (Table S3), 37 were differentially expressed between metastatic and primary tumors in the GEO‐OV dataset as shown in Figure 1B. Of these, CXCL12 exhibited the highest fold change in expression between metastatic and primary tumors (Figure 6E). In the TCGA‐OV dataset, only CXCL12 and ACKR4 showed positive correlations with both GALNT10 and IGFBP7 (Figure 6F,G; Figure S12B,C), supporting their selection as key chemokines.

To detect immune infiltration in tumor tissues, we injected luciferase‐labeled ID8 cells into the ovarian bursa of C57 mice to establish the orthotopic ovarian cancer model. Three weeks after injection, the mice were divided into the Luteolin treatment group and the control group. The mice in the treatment group received intraperitoneal injection of 50 mg/kg Luteolin every 3–4 days, and tumor metastasis was monitored by bioluminescent imaging (Figure 6H). After 6 weeks of treatment, Luteolin markedly reduced peritoneal metastasis compared with the control group (Figure 6I; Figure S13A). In Luteolin‐treated tumors, qPCR and IHC showed reduced CXCL12 versus the control group (Figure S12D–F), confirming its role in immune regulation. CyteFlow analysis of tumor tissues revealed a significant increase in CD8+ T cell and CD4+ T cell infiltration in the Luteolin group (Figure 6J–N). Therefore, we further explored whether Luteolin exhibits a synergistic effect with immunotherapy. Luciferase‐labeled ID8 cells were intraperitoneally injected to establish an ovarian cancer peritoneal metastasis model. The C57 mice were divided into four groups 3 weeks after injection: Luteolin, anti‐PD1, combined treatment, and control. One week later, bioluminescent imaging demonstrated that the combined therapy group exhibited a superior inhibitory effect on metastasis compared to the single‐drug groups (Figure 6O,P; Figure S13B). These results indicate that the GALNT10 inhibitor Luteolin promotes immune infiltration and has a synergistic effect with anti‐PD1.

## 3 Discussion

Tumor metastasis is a clinical challenge of ovarian cancer, necessitating the urgent development of novel therapeutic strategies. In this study, we demonstrate that glycosylation supports ovarian cancer metastasis, with GALNT10 acting as a key glycosyltransferase. Mechanistically, GALNT10 plays a dual role by regulating EMT in a cell‐autonomous manner and modulating the vasculature and immune microenvironment through non‐cell‐autonomous mechanisms. Furthermore, we identified Luteolin as a potent GALNT10 inhibitor capable of effectively suppressing ovarian cancer metastasis. Notably, Luteolin exhibits synergistic effects when combined with anti‐PD1 therapy.

Glycosylation offers a novel perspective for elucidating the mechanisms of ovarian cancer metastasis. Tumor metastasis is fundamentally a process involving interactions between tumor cells and the microenvironment, mediated by multiple membrane proteins and secreted proteins. Notably, over 70% of these proteins undergo glycosylation [27], which plays a critical role in tumor metastasis [11]. There are two major types of glycosylation, N‐glycosylation and O‐glycosylation. N‐glycosylation occurs when the sugar chain is attached to the NH_2_ group of asparagine residues. The sugar chain in N‐glycosylation usually comprises a pentasaccharide core with various branching structures. In contrast, O‐glycosylation involves the attachment of the sugar chain to the OH group of serine or threonine residues [9]. Unlike N‐glycosylation, O‐glycosylated sugar chains do not possess a conserved core structure and are typically synthesized through the sequential addition of monosaccharides. Cancer exhibits extensive glycosylation‐related alterations, including truncated O‐glycans, increased sialylation, increased branching structures, and overexpression of fucosylation, which interfere with the cell‐cell adhesion mediated by epithelial cadherins, regulate the interaction between tumor cells and the extracellular matrix, and promote the detachment and migration invasion processes of tumor cells [11].

Studies have demonstrated that alterations in O‐glycosylation significantly contribute to tumor invasion and metastasis. Increased O‐glycosylation of MMP14 promotes liver cancer cell metastasis [28], and abnormal O‐glycosylation of MUC1 activates the PI3K/AKT pathway, thereby driving the progression and metastasis of colon cancer [29]. Additionally, O‐glycosylation of Galectin‐4 mediates EGFR activation and downstream signaling pathways, inducing EMT of prostate cancer cells and facilitating metastasis [30]. In this study, we found that one of the O‐glycosylation, O‐GalNAc, is critical in ovarian cancer metastasis. The GALNTs family is closely associated with tumor development [11], and numerous studies have confirmed the critical role of GALNT10 in tumor progression. GALNT10 has been identified as a marker of poor prognosis in clear cell renal cell carcinoma [31]. GALNT10 promotes the growth of liver cancer cells by activating the EGF signaling pathway through glycosylation [32]. Additionally, GALNT10 promotes the proliferation, metastasis, and chemotherapy resistance of gastric cancer cells by regulating the expression of HOXD13 [33]. In ovarian cancer, GALNT10 has been implicated in maintaining cancer stemness and contributing to an immunosuppressive microenvironment [34, 35], although the precise mechanisms were previously unclear. This study confirms that GALNT10 is significantly associated with metastasis and poor prognosis in ovarian cancer, based on integrated multi‐omics analysis and supported by functional experiments. Moreover, the regulatory mechanism of GALNT10 was thoroughly investigated via glycoproteomics.

IGFBP7 is a secreted protein, one of the members of the IGFBP family. This family binds insulin and insulin‐like growth factors (IGFs) through the N‐terminal domain, thereby regulating IGF binding to their receptors and influencing cell proliferation, apoptosis, adhesion, and other processes [21]. However, the key residues in IGFBP7 for IGF binding are not conserved, resulting in its relatively low binding affinity to IGF. Consequently, IGFBP7 predominantly exerts its functions in an IGF‐independent manner. A study confirmed that IGFBP7 promotes tumor progression by inducing vascular homeostasis imbalance [23]. Prior to our work, it was unknown how glycosylation of IGFBP7 influences its function. Here, we show that GALNT10‐mediated O‐GalNAc glycosylation of IGFBP7 at the T188 site regulates its secretion and function.

Angiogenesis has been recognized as a core hallmark of cancer growth and survival. Tumor vasculatures are typically tortuous, with poorly organized pericyte coverage, hyperdilated and leaky, with aberrant blood flow hindering drug and oxygen delivery while supporting tumour cell metastasis [36]. An increasing number of studies have shown that the tumor vascular‐immune crosstalk has a significant impact on tumor progression and the efficacy of immunotherapy [37, 38]. Furthermore, we identified CXCL12 as a key chemokine potentially regulated by GALNT10, which has been reported to be induced by hypoxia and facilitate the differentiation and infiltration of regulatory T cells, tumor‐associated macrophages, and myeloid‐derived suppressor cells, thereby contributing to the establishment of an immunosuppressive tumor microenvironment. [39, 40] A recent study has demonstrated that CXCL12 promotes epithelial‐mesenchymal transition in colorectal cancer cells while concurrently suppressing CD8^+^ T cell infiltration [41]. These observations align with the results of the present study, which indicate that the GALNT10‐IGFBP7 axis contributes to immunosuppressive microenvironment formation through CXCL12 signaling.

Targeting vascular normalization has emerged as a promising strategy to improve vascular‐immune crosstalk and works in synergy with chemotherapy and immunotherapy [42, 43, 44]. Besides, targeting O‐glycosylation can modulate cancer and immune cell crosstalk and enhance anti‐PD‐1 therapy in head and neck cancer [45]. Our findings demonstrate that Luteolin, a GALNT10 inhibitor, effectively suppresses metastasis and promotes vascular normalization, exhibiting synergistic effects with anti‐PD1 therapy. As a natural compound, Luteolin is widely present in various plant species and has been utilized as a key ingredient in health supplements due to its antioxidant, anti‐inflammatory, and neuroprotective properties, which collectively demonstrate a favorable safety profile. In recent years, its anti‐cancer potential has attracted increasing attention, with studies reporting anti‐tumor activities in breast cancer, colorectal cancer, and lung cancer [46]. These findings suggest that Luteolin has the potential to serve as a novel therapeutic agent for the treatment of ovarian cancer.

## 4 Conclusion

Overall, our findings demonstrated that glycosylation exerts regulatory effects not only in tumor cells but also within cellular interactions in the tumor microenvironment, and further elucidated the critical role of the GALNT10‐IGFBP7 axis in vascular remodeling, immune crosstalk, and tumor metastasis in ovarian cancer. Moreover, we identified the GALNT10 inhibitor Luteolin, which effectively suppresses ovarian cancer metastasis, modulates the immunosuppressive tumor microenvironment, and exhibits synergistic effects with anti‐PD1 therapy. Our study offers novel therapeutic perspectives for ovarian cancer metastasis and for immunotherapy sensitization.

## 5 Experimental Section

### 5.1 Reagents and Antibodies

Luteolin was purchased from MedChemExpress (HY‐N0162). Ac_5_GalNTGc was purchased from Biosynth Carbosynth (M145185), Benzyl‐α‐GalNAc was purchased from MedChemExpress (HY‐129389), and Urolithin D was purchased from Topscience (T74005). All of those compounds were solubilized to 10 mmol/L in DMSO for in vitro assays. Anti‐mouse PD1 in vivo was purchased from Selleck (A2122). Antibodies used for immunoblotting, immunoprecipitation, and CyteFlow analysis were summarized in Supplementary Table S4. All siRNAs were ordered from JTSBIO Co., Ltd (Wuhan, China), all of the shRNA was ordered from Mailgene biosciences Co., Ltd. All siRNA and shRNA sequences were summarized in Supplementary Table S5. All solvents and reagents were used as received without further purification unless specified.

### 5.2 Cell Lines

The sources of cell lines and cell cultures were summarized in Supplementary Table S6. All cell lines were routinely authenticated through short tandem repeat (STR) DNA fingerprinting routinely. The cell lines were contamination‐free. Mycoplasma contamination was excluded using the MycoBlue Mycoplasma Detector (Vazyme Biotech, Nanjing, China).

### 5.3 Clinical Specimen

Tissue microarrays were constructed by Li΄s laboratory [47], one of which contains 48 paired metastasis and primary tumor of ovarian cancer, the other one contains tumor tissues of 119 patients with ovarian cancer who underwent cytoreductive surgery and platinum‐based chemotherapy. The clinical characteristics of patients were collected from medical records. Overall survival (OS) refers to the date of surgery to the end of follow‐up (November 2019) or the date of death. Besides, the 119 patients were divided into the GALNT10^high^ group and GALNT10^low^ group based on the median of GALNT10 intensity values according to the immunohistochemistry. Survival analysis was performed by the Kaplan‐Meier method. The experiment was approved by the Medical Science Research Ethics Committee of Peking University Third Hospital (Approval ID: S20250351), and the informed written consent of all participants was obtained.

### 5.4 Retrieval of Metabolic Pathway Gene Sets and GSVA Enrichment Analysis

The R package KEGGREST was used to retrieve all human metabolic pathways and extract the gene lists associated with each pathway. For GSVA, default parameters were applied (method: gsva, kernel: Gaussian) to transform the batch‐corrected and integrated gene expression matrix of ovarian cancer datasets (GSE218939, GSE62873, and GSE138866) into a pathway activity matrix, quantifying the enrichment level of each sample in metabolic pathways. The Wilcoxon rank‐sum test was then used to identify differentially enriched metabolic pathways between primary and metastatic ovarian cancers. Survival analysis was performed using the survival and survminer R packages. Patients were stratified into high‐ and low‐expression groups based on the median gene expression level. Kaplan‐Meier survival curves were then constructed, and differences between groups were assessed using the log‐rank test (with a significance threshold of *p* < 0.05). Finally, survival curves were visualized using the ggsurvplot function.

### 5.5 Immunohistochemistry (IHC)

IHC was performed with primary antibodies against GALNT10 (1:200, Abcam, ab106471), CD31(1:300, Servicebio, GB113151) and α‐SMA (1:1000, Servicebio, GB113151) according to the manufacturers’ recommendations. Briefly, the slides were baked at 60°C overnight and subsequently dewaxed in xylene. Dehydration was carried out by an alcohol gradient of 95%, 85%, and 70%. Antigen retrieval was conducted by heating slides immersed in Tris/EDTA buffer pH 9.0. After staining, the tissue sections were scanned and digitized with Aperio's ImageScope software (Aperio, Vista, CA, USA). The staining intensity was evaluated and categorized as “0” (negative), “1” (weakly stained), “2” (moderately stained), or “3” (strongly stained). The proportion of positive cells was also scored. And the final IHC staining score was determined by multiplying the intensity score by the percentage score.

### 5.6 Cell viability assays

For cell viability, 1 × 10^4^ ovarian cancer cells were seeded into 96‐well microplates per well, and cell viability was detected by CCK8 every day for 4 days. For the drug IC50 test, cells were incubated with drugs for 24 h after cell adherence, and then cell viability was detected by CCK8. Cell viability was determined by normalizing the absorbance at 450 nm of the experimental groups to that of the negative control group. The data are presented as the mean ± standard deviation (SD).

### 5.7 Clone Formation

1 × 10^3^ ovarian cancer cells were seeded into each well of the 6‐well microplates and cultured for approximately 2 weeks, with the culture medium replaced every 2–3 days during this period. The culture was terminated when clusters of cells appeared as clones. The cells were then fixed with 4% paraformaldehyde and stained with 0.5% crystal violet at room temperature for 30 min. Following staining, the excess crystal violet was removed, and the clones were photographed.

### 5.8 Transwell

650 µL of 10% FBS culture medium was added to each well of the 24‐well plate under the Transwell chamber. And then (2‐5) × 10^4^ ovarian cancer cells or 5 × 10^4^ HUVECs suspended in 100 µL of FBS‐free culture medium were seeded into the Transwell chamber. After 16 h, the Transwell chambers were fixed in 4% paraformaldehyde and stained with 0.5% crystal violet at room temperature for 30 min. Cells remaining on the upper surface of the chamber were removed, and the transwelled cells were photographed under a microscope.

### 5.9 Wound‐Healing Assay

8 × 10^5^ ovarian cancer cells were seeded into each well of the 6‐well microplates. After the cell attachment reached approximately 90% confluence, a wound was created on the cell monolayer by manually scraping with a p200 pipette tip. The initial image was captured as a reference. The cells were then incubated in a 2% FBS culture medium for 24–48 h, and images were acquired for comparison.

### 5.10 Glycoproteomics

Glycoproteomic analysis was performed on GALNT10 knockdown (shGALNT10) and on negative control (shNC) SKOV3 cells. Through a series of cutting‐edge technologies such as protein extraction, enzymatic digestion, enrichment of modified peptides, liquid chromatography‐tandem mass spectrometry analysis, and bioinformatics analysis, the modified quantitative proteomics of the samples is studied. For detailed information, please refer to the Supplementary Material.

### 5.11 Co‐Immunoprecipitation (Co‐IP) and Western Blot

Cell pellets were lysed with RIPA lysis buffer supplemented with a protease inhibitor cocktail (Roche, Basel, Switzerland) for 40 min on ice. Following lysis, the samples were centrifuged at 12 000 g for 15 min. The total protein in the supernatants was collected, and concentrations were determined using a BCA kit (Thermo Scientific). For Co‐IP, protein A/G magnetic beads (HY‐K0202, MCE) were incubated with anti‐GALNT10 antibody for 2 h at 4 °C. The antibody‐conjugated magnetic beads were then incubated with equal amounts of cell lysate at 4 °C overnight. The beads were washed 3 times with lysis buffer, and the immunoprecipitated proteins were analyzed by Western blot. β‐Actin was used as an endogenous control. Western blot analysis was conducted according to standard protocols.

### 5.12 VVL Blot

Sample preparation, electrophoresis, and membrane transfer are the same as in Western Blot. The membrane was blocked using the lectin blot blocking solution (S‐9005, Vector Labs) at room temperature for 40 min, and then incubated with a 1:1000 dilution of VVL (B‐1235‐2, Vector Labs) overnight at 4°C. After being washed with TBST 3 times, the membrane was then incubated with a 1:2000 dilution of HRP‐conjugated Streptavidin at room temperature for 2 h. The chemiluminescent assay was performed in the same way as a Western blot.

### 5.13 Tube Formation

The HUVECs were incubated with the culture supernatant from OE‐IGFBP7 cells, shGALNT10+OE‐IGFBP7 cells, and control cells for 24 h. 60 µL of Matrigel was added to each well of the 96‐well microplates and incubated at 37°C for 1 h. The HUVECs were then digested and then seeded into the Matrigel‐coated 96‐well plate. After incubation at 37°C for 6 h, the wells were photographed under a microscope.

### 5.14 Immunofluorescence Staining

5 × 10^4^ ovarian cancer cells were seeded into each well of the 6‐well microplates. Following cell adherence, the cells were fixed with 4% paraformaldehyde and permeabilized with Triton X‐100. Subsequently, the cells were incubated with antibodies against GALNT10 (red), IGFBP7/Fibulin3/Agrin (green), and DAPI‐stained nuclei (blue), after which they were visualized and digitized under a fluorescence microscope.

### 5.15 CyteFlow Analysis

0.5–1 g of mouse ovarian cancer tissue was thoroughly minced into small pieces and subsequently digested in 4 mL of tissue digestion solution at 37 °C with shaking at 160 rpm for 1 h. The mixture was gently vortexed every 15 min for 20–30 s to ensure uniform digestion. Following digestion, the cell suspension was filtered through a 300‐mesh nylon mesh to remove undigested debris. The cells were then incubated with red blood cell lysis buffer at room temperature for 5 min to eliminate contaminating erythrocytes. The lysis reaction was terminated by adding an equal volume of PBS. The cell suspension was centrifuged at 500 g for 5 min at 4 °C to collect the cell pellet, which was subsequently resuspended in PBS to prepare a single‐cell suspension. The antibody cocktail was pre‐diluted in PBS at a ratio of 1:20. Then, 50 µL of the cell suspension was transferred into a new tube and incubated with 5 µL of the pre‐diluted antibody at room temperature in the dark for 15 min to allow antibody binding. The stained cells were washed with 200 µL of PBS to remove unbound antibodies and then analyzed by flow cytometry.

### 5.16 Animal Xenograft and Treatments

All animal protocols were approved by the Animal Care and Use Committee of Peking University Third Hospital (Approval ID: SA20250338). 5 × 10^5^ A2780‐luc or ID8‐luc cells were injected into the bursa of the right ovary of five‐week‐old female BALB/c nude mice or C57 mice (Beijing Vital River Laboratory Animal Technology Co., Ltd., Beijing, China) to establish an orthotopic ovarian tumor model. Additionally, 5 × 10^6^ SKOV3‐luc or ID8‐luc cells were intraperitoneally injected to establish a peritoneal metastasis model. Following completion of the experiment, blood samples were collected from the orbital sinus of the mice to assess serological indicators of cardiac, hepatic, and renal function. The tumor tissues and major organs were then harvested for dissection and subsequently subjected to IHC or HE staining.

### 5.17 RNA Extraction and RT‐qPCR

Total RNA was extracted from orthotopic tumor tissue as indicated using RNA Easy Fast Tissue/Cell Kit (DP451, TIANGEN), and then reverse transcribed into cDNA using a FastKing RT Kit (KR116, TIANGEN). qRT‐PCR was performed on a StepOnePlus Real‐Time PCR system using standard procedures. The relative expression levels of chemokine genes were normalized to GAPDH as an endogenous control. Primers are listed in Table S7.

### 5.18 Statistical Analysis

Data are shown as mean ± SD represented by at least three repeated experiments. Statistical analysis was performed by two‐tailed, unpaired Student's t‐test or Mann‐Whitney U test with SPSS 26.0 software. Survival analysis was performed by the Kaplan‐Meier method. The graphs were plotted using GraphPad Prism 8.0 software.

## Author Contributions

Y.Z. contributed to conception, data curation, formal analysis, funding acquisition, investigation, methodology, and drafted and critically revised the manuscript. A.Z. contributed to data acquisition, formal analysis, and investigation, and revised the manuscript. Z.W. contributed to data curation, formal analysis, and investigation. A.L. contributed to methodology, supervision, and revised the manuscript. B.J. and Q.S. contributed to data analysis and methodology. Y.L. contributed to supervision and validation. Q.L. contributed to formal analysis, supervision, and validation. H.G. contributed to funding acquisition, supervision, validation, and revised the manuscript. C.S. contributed to conceptualization, funding acquisition, methodology, supervision, and revised the manuscript.

## Funding

This work was supported by the National Natural Science Foundation of China (82504130), the China Postdoctoral Science Foundation (Certificate Number: 2024M760142), the State Key Laboratory of Vascular Homeostasis and Remodeling Open Funding (2025‐VHR‐O‐SY‐17), the National Natural Science Foundation of China (82273383), Peking University Clinical Scientist Training Program, supported by “the Fundamental Research Funds for the Central Universities”, the Key Clinical Project of Peking University Third Hospital (BYSY2022050), the National Key Research and Development Program of China (2022YFC2704000), the Clinical Medicine Plus X—Young Scholars Project, the Peking University (PKU2022LCXQ020) and the young talent lifting project organized by Beijing Association of Science and Technology, the Ningxia Key Research and Development Program (2023BEG01001).

## Conflicts of Interest

The authors declare no conflicts of interest.

## Supporting information




**Supporting File 1**: advs74014‐sup‐0001‐SuppMat.docx.

**Supporting File 2**: advs74014‐sup‐0002‐Data.zip.

**Supporting File 3**: advs74014‐sup‐0003‐FigureS1‐S13.docx.

**Supporting File 4**: advs74014‐sup‐0004‐TableS1‐S7.xlsx.

## Data Availability

The data that support the findings of this study are available from the corresponding author upon reasonable request.
