# Supplementary material for: GALNT10 Affects O‐Glycosylation of IGFBP7 to Promote Tumor Vascular Remodeling and Metastasis of Ovarian Cancer
Source: Adv Sci (Weinh). 2026 Feb 4;13(19):e16106. doi: 10.1002/advs.202516106 (PMC13045431; doi:10.1002/advs.202516106)
Supplement: Supplementary file 1 — Supporting File 1: advs74014‐sup‐0001‐SuppMat.docx. [file ADVS-13-e16106-s001.docx]

**Protocol of Glycoproteomics**


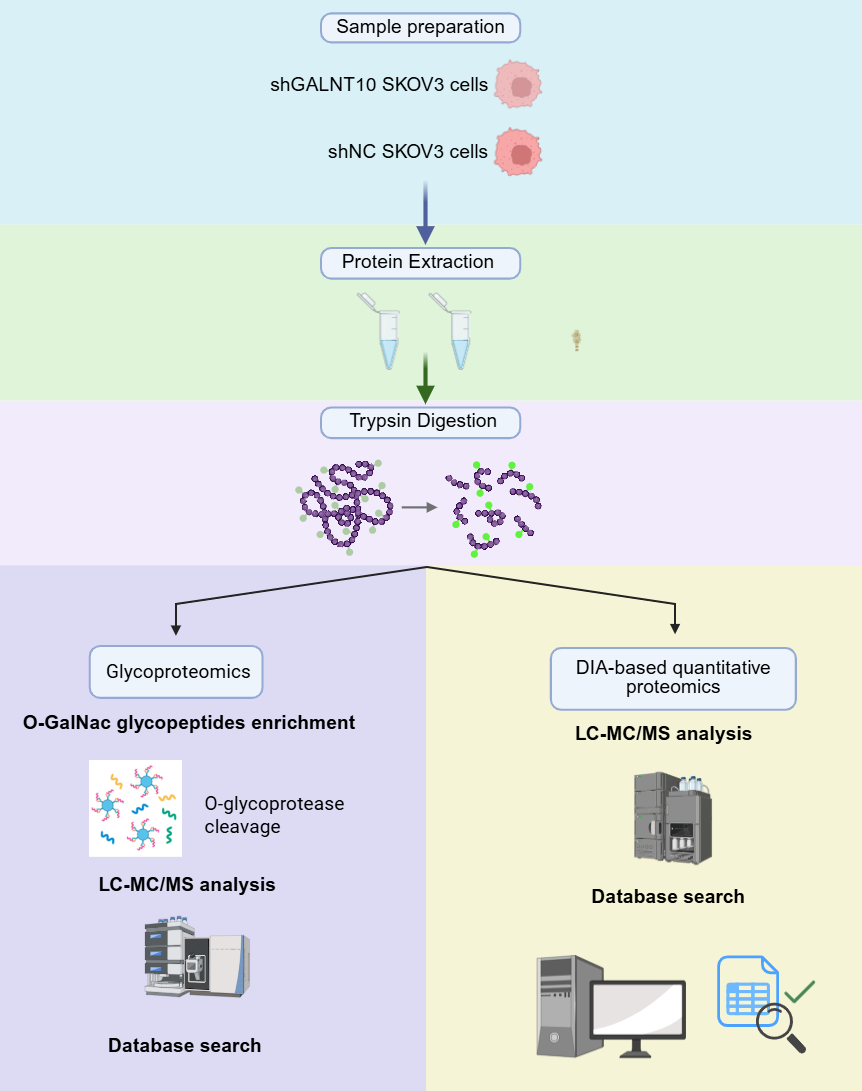


**The flowchart of glycoproteomics and quantitative proteomics.**

**1. Sample Preparation**

2×10^7^ shGALNT10 and shNC SKOV3 cells were collected respectively, and the cell pellets were transferred into centrifuge tubes and rapidly frozen at -80°C for subsequent analysis.

**2. Protein Extraction**

Samples were sonicated for three minutes on ice using a high intensity ultrasonic processor (Scientz) in lysis buffer (8 M urea, 1% protease inhibitor cocktail). The remaining debris was removed by centrifugation at 12,000 g at 4 °C for 10 min. Finally, the supernatant was collected and the protein concentration was determined with BCA kit according to the manufacturer’s instructions.

**3. Trypsin Digestion**

The sample was slowly added to the final concentration of 20% (m/v) TCA to precipitate protein, then vortexed to mix and incubated for 2 h at 4 °C. The precipitate was collected by centrifugation at 4500 g for 5 min at 4 °C. The precipitated protein was washed twice with pre-cooled acetone and dried for 1 min. The protein sample was then redissolved in 200 mM TEAB and ultrasonically dispersed. Trypsin was added at 1:50 trypsin-to-protein mass ratio for the first digestion overnight. The sample was reduced with 5 mM dithiothreitol for 60 min at 37 °C and alkylated with 11 mM iodoacetamide for 45 min at room temperature in darkness. Finally, the peptides were desalted by Strata X SPE column.

**4. Glycoproteomics Analysis**

4.1 Bio-material-based PTM enrichment (for O-GalNac)

Tryptic peptides dissolved in guanidination solution were incubated at 65°C for 20 min. Following this incubation, the solution was cooled to RT and loaded on a pre-conditioned C18 column. The column was then washed three times with 0.1% TFA and peptides were eluted in 60% acetonitrile/0.1% TFA. Intact glycopeptides were enriched using a SAX HyperSepTM Retain AX Columns (RAX). The eluted intact glycopeptides were then incubated with the resin (100 μg/100 μl resin, 50% slurry) and 50 mM sodium cyanoborohydride (NaCNBH3) at RT overnight with shaking. The resin was washed three times with 50% acetonitrile, 1.5 M NaCl, and 20 mM Tris-HCl buffer (pH 6.8). O-linked glycopeptides were released from the resin by incubation with O-glycoprotease in 100 μL of 20 mM Tris-HCl buffer (pH 6.8) at 37°C for 16 h according to the manufacturer’s instructions. Following this 16-h incubation, the released peptides in solution were collected. For LC-MS/MS analysis, the resulting peptides were desalted with C18 ZipTips (Millipore) according to the manufacturer’s instructions.

4.2 LC-MS/MS Analysis for glycoproteomics

The tryptic peptides were dissolved in solvent A, directly loaded onto a home-made reversed-phase analytical column (15-cm length, 100 μm i.d.). The mobile phase consisted of solvent A(0.1% formic acid in water) and solvent B (0.1% formic acid, 80% acetonitrile/in water). Peptides were separated with the following gradient:0-0.75 min, 4.0%B; 0.75-0.90 min, 4.0%-8.0%B; 0.90-1.35 min, 8.0%-8.5%B; 1.35-20.85 min, 8.5%-22.5%B; 20.85-31.35 min, 22.5%-35%B; 31.35-31.95 min, 35.0-55.0%B; 31.95-32.70 min, 55.0-99.0%B; 32.70-34.00 min, 99.0%B, and all at a constant flow rate of 400 nl/min on a Vanquish Neo UPLC system (ThermoFisher Scientific).

The separated peptides were analyzed in Orbitrap Astral with a nano-electrospray ion source. The electrospray voltage applied was 1900 V. Precursors were analyzed at the Orbitrap detector, and the fragments were analyzed at the Astral detector. The full MS scan resolution was set to 240000 for a scan range of 350-1800 m/z. The first mass of the MS/MS scan was fixed at 120.0 m/z at a resolution of 80,000. The Cycle Time was set as 0.6 s. Automatic gain control (AGC) target was set at 100%, with an intensity threshold of 2500 ions/s and a maximum injection time of 5 ms.

4.3 Database search

Raw data were processed using MSFragger (v.3.4) software, tandem mass spectra were searched against the Homo_sapiens_9606_SP_20231220.fasta (20429 entries) concatenated with reverse decoy database. Enzymes were set to stricttrypsin and N-terminal of ST, missed cleavages was set to 2 and 5, respectively. The length range of the peptide was set to 7-50. Carbamidomethyl on Cys was specified as fixed modification. Acetylation on protein N-terminal, oxidation on Met were specified as variable modifications. Mass offsets were set to the default list of glycosylated modifications. False discovery rates (FDRs) of proteins, peptides, and PSMs were adjusted to < 1%.

**5. Quantitative Proteomics**

5.1 LC-MS/MS Analysis for DIA (Data independent acquisition) quantitative proteomics

The tryptic peptides were dissolved in solvent A, directly loaded onto a home-made reversed-phase analytical column (15-cm length, 100 μm i.d.). The mobile phase consisted of solvent A(0.1% formic acid in water) and solvent B (0.1% formic acid, 80% acetonitrile/in water). Peptides were separated with the following gradient: 0-1.6 min, 4%-22.5%B; 1.6-2.0 min, 22.5%-35%B; 2.0-2.6 min, 35%-55%B; 2.6-2.7 min, 55%-99%B; 2.7-6.8 min, 99%B; 6.8-7.6 min, 99%B, and all at a constant flow rate of 300 nl/min on a Vanquish Neo UPLC system (ThermoFisher Scientific).

The separated peptides were analyzed in Orbitrap Astral with a nano-electrospray ion source. The electrospray voltage applied was 1900 V. Precursors were analyzed at the Orbitrap detector, and the fragments were analyzed at the Astral detector. The full MS scan resolution was set to 240000 for a scan range of 480-780 m/z. The first mass of the MS/MS scan was fixed at 150.0 m/z at a resolution of 80,000. The HCD fragmentation was performed at a normalized collision energy (NCE) of 25 %. Automatic gain control (AGC) target was set at 500%, with a maximum injection time of 3 ms.

5.2 Database search

The DIA data were processed using the DIA-NN search engine (v.1.8). Tandem mass spectra were searched against the Homo_sapiens_9606_SP_20231220.fasta (20429 entries) concatenated with reverse decoy database. Trypsin/P was specified as cleavage enzyme allowing up to 1 missing cleavages. Excision on N-term Met and carbamidomethyl on Cys were specified as fixed modification. FDR was adjusted to < 1%.
