# Supplementary material for: GALNT10 Affects O‐Glycosylation of IGFBP7 to Promote Tumor Vascular Remodeling and Metastasis of Ovarian Cancer
Source: Adv Sci (Weinh). 2026 Feb 4;13(19):e16106. doi: 10.1002/advs.202516106 (PMC13045431; doi:10.1002/advs.202516106)
Supplement: Supplementary file 2 — Supporting File 2: advs74014‐sup‐0002‐Data.zip. [file ADVS-13-e16106-s003.zip › advs74014-sup-0002-Data/Original images of blots.pdf]

Figure S3

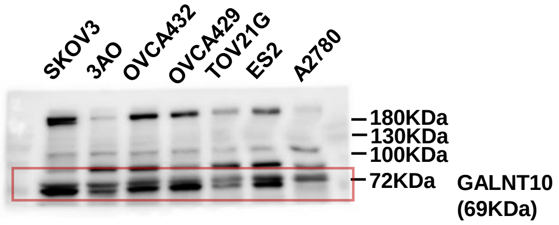
SKOV3
3AO
OVCA432
OVCA429
TOV21G
ES2
A2780
180KDa
130KDa
100KDa
72KDa
GALNT10
(69KDa)

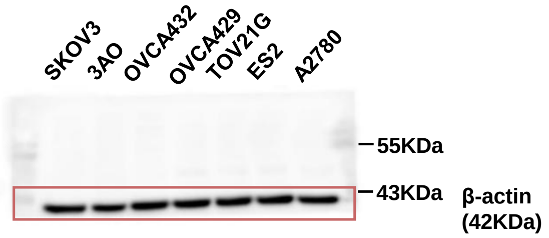
SKOV3
3AO
OVCA432
OVCA429
TOV21G
ES2
A2780
55KDa
43KDa
β-actin
(42KDa)

Figure 2A

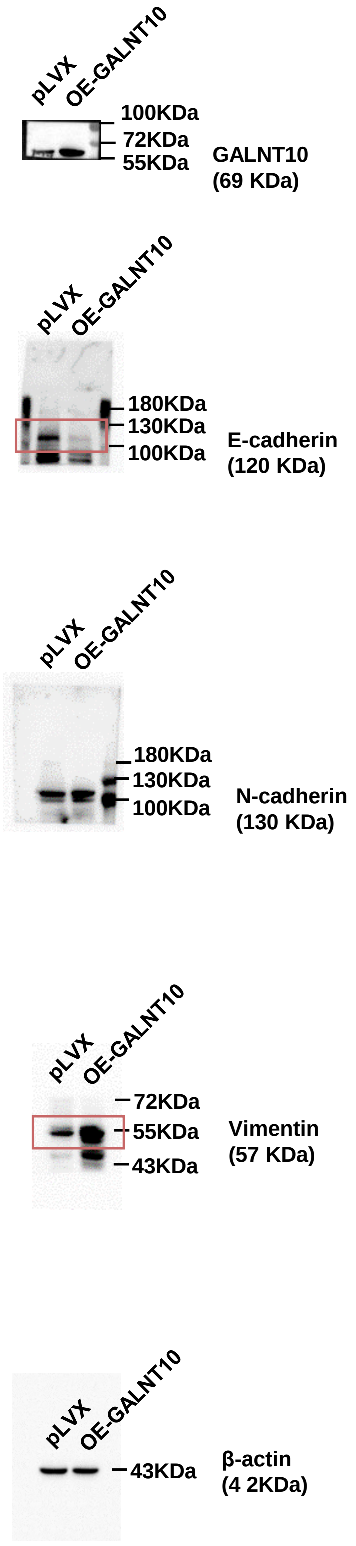
pLVX
OE-GALNT10
100KDa
72KDa
55KDa
GALNT10
(69 KDa)
pLVX
OE-GALNT10
180KDa
130KDa
100KDa
E-cadherin
(120 KDa)
pLVX
OE-GALNT10
180KDa
130KDa
100KDa
N-cadherin
(130 KDa)
pLVX
OE-GALNT10
72KDa
55KDa
43KDa
Vimentin
(57 KDa)
pLVX
OE-GALNT10
43KDa
β-actin
(4 2KDa)

Figure 2B

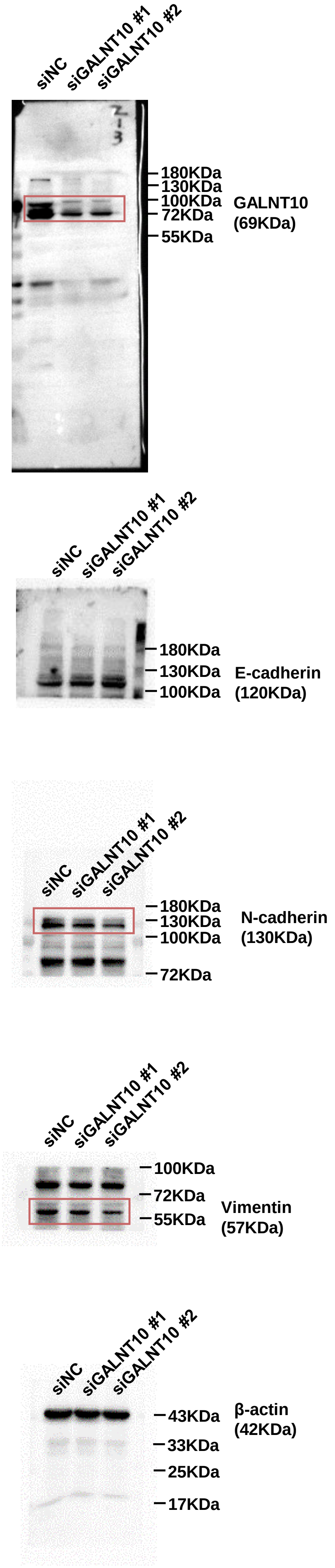
siNC
siGALNT10 #1
siGALNT10 #2
180KDa
130KDa
100KDa
72KDa
55KDa
GALNT10
(69KDa)
siNC
siGALNT10 #1
siGALNT10 #2
180KDa
130KDa
100KDa
E-cadherin
(120KDa)
siNC
siGALNT10 #1
siGALNT10 #2
180KDa
130KDa
100KDa
72KDa
N-cadherin
(130KDa)
siNC
siGALNT10 #1
siGALNT10 #2
100KDa
72KDa
55KDa
Vimentin
(57KDa)
siNC
siGALNT10 #1
siGALNT10 #2
43KDa
33KDa
25KDa
17KDa
β-actin
(42KDa)

Figure 2G

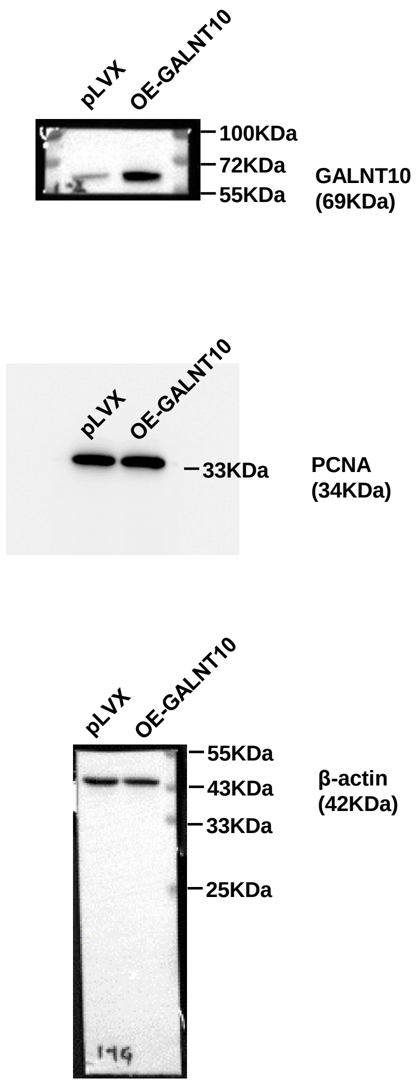
pLVX
OE-GALNT10
100KDa
72KDa
55KDa
GALNT10
(69KDa)
pLVX
OE-GALNT10
33KDa
PCNA
(34KDa)
pLVX
OE-GALNT10
55KDa
43KDa
33KDa
25KDa
β-actin
(42KDa)

Figure 2H

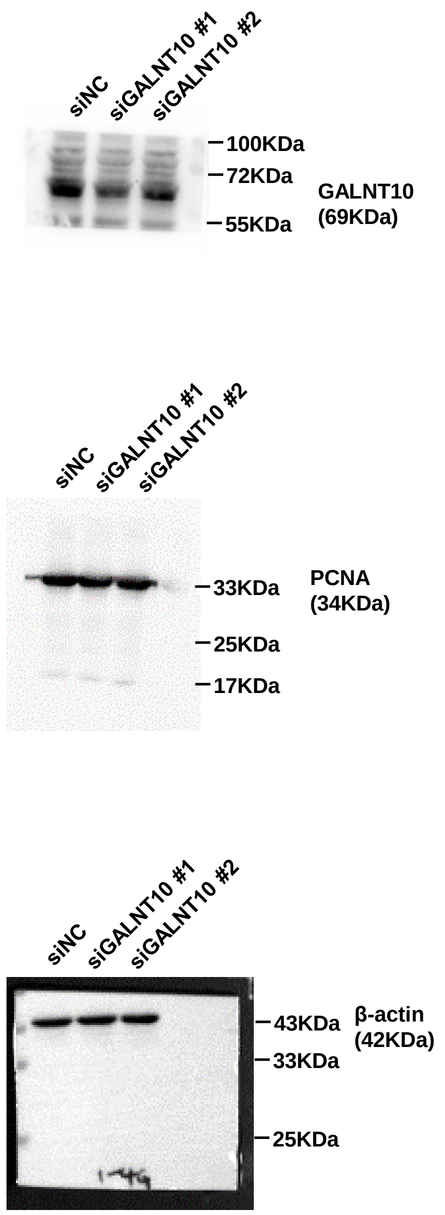
siNC
siGALNT10 #1
siGALNT10 #2
100KDa
72KDa
55KDa
GALNT10
(69KDa)
siNC
siGALNT10 #1
siGALNT10 #2
33KDa
25KDa
17KDa
PCNA
(34KDa)
siNC
siGALNT10 #1
siGALNT10 #2
43KDa
33KDa
25KDa
β-actin
(42KDa)

Figure 3A

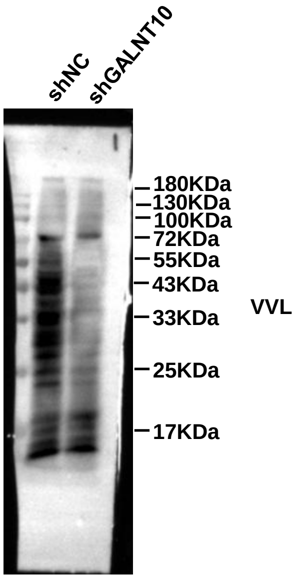
shNC
shGALNT10
180KDa
130KDa
100KDa
72KDa
55KDa
43KDa
33KDa
25KDa
17KDa
VVL

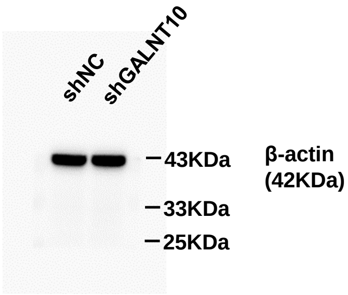
shNC
shGALNT10
43KDa
33KDa
25KDa
β-actin
(42KDa)

Figure 3E

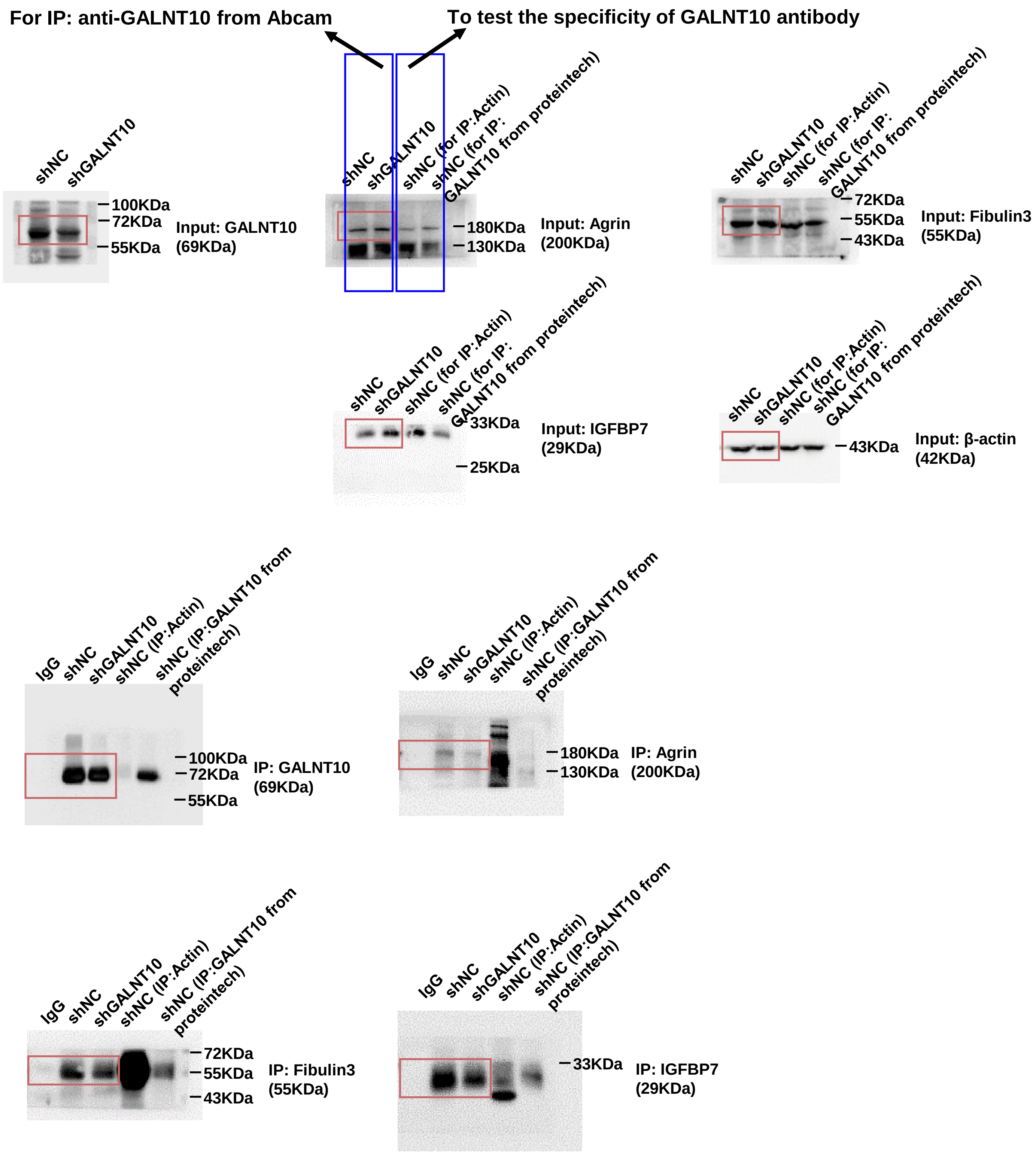
For IP: anti-GALNT10 from Abcam
To test the specificity of GALNT10 antibody
shNC
shGALNT10
100KDa
72KDa
55KDa
Input: GALNT10
(69KDa)
shNC
shGALNT10
shNC (for IP:Actin)
shNC (for IP:
GALNT10 from proteintech)
180KDa
130KDa
Input: Agrin
(200KDa)
shNC
shGALNT10
shNC (for IP:Actin)
shNC (for IP:
GALNT10 from proteintech)
72KDa
55KDa
43KDa
Input: Fibulin3
(55KDa)
shNC
shGALNT10
shNC (for IP:Actin)
shNC (for IP:
GALNT10 from proteintech)
33KDa
25KDa
Input: IGFBP7
(29KDa)
shNC
shGALNT10
shNC (for IP:Actin)
shNC (for IP:
GALNT10 from proteintech)
43KDa
Input: β-actin
(42KDa)
IgG
shNC
shGALNT10
shNC (IP:Actin)
shNC (IP:GALNT10 from
proteintech)
100KDa
72KDa
55KDa
IP: GALNT10
(69KDa)
IgG
shNC
shGALNT10
shNC (IP:Actin)
shNC (IP:GALNT10 from
proteintech)
180KDa
130KDa
IP: Agrin
(200KDa)
IgG
shNC
shGALNT10
shNC (IP:Actin)
shNC (IP:GALNT10 from
proteintech)
72KDa
55KDa
43KDa
IP: Fibulin3
(55KDa)
IgG
shNC
shGALNT10
shNC (IP:Actin)
shNC (IP:GALNT10 from
proteintech)
33KDa
IP: IGFBP7
(29KDa)

Figure 3L

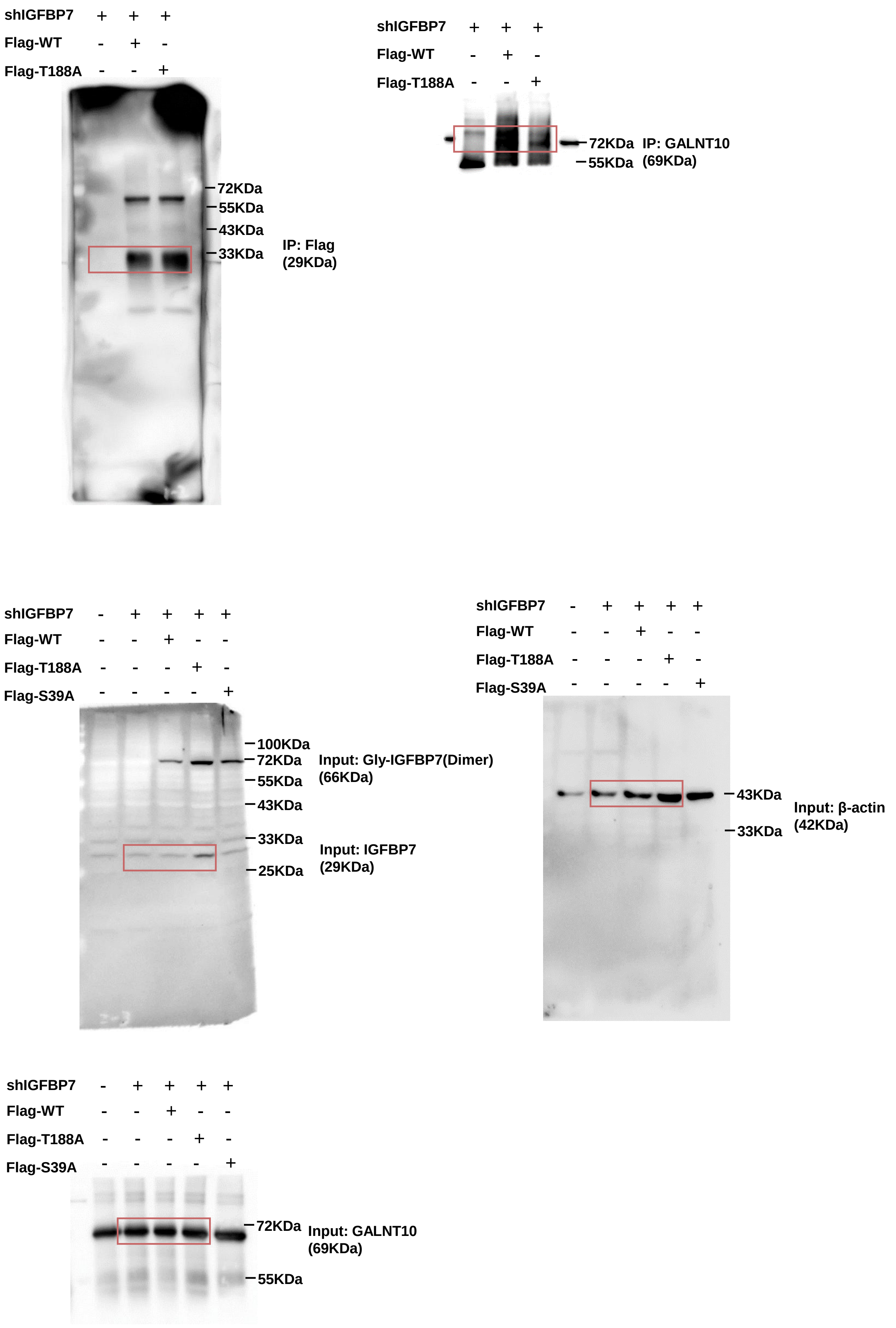

shIGFBP7 + + +
Flag-WT - + -
Flag-T188A - - +
72KDa
55KDa
43KDa
33KDa
IP: Flag
(29KDa)
shIGFBP7 + + +
Flag-WT - + -
Flag-T188A - - +
72KDa
55KDa
IP: GALNT10
(69KDa)
shIGFBP7 - + + + +
Flag-WT - - + - -
Flag-T188A - - - + -
Flag-S39A - - - - +
100KDa
72KDa
55KDa
43KDa
33KDa
25KDa
Input: Gly-IGFBP7(Dimer)
(66KDa)
Input: IGFBP7
(29KDa)
shIGFBP7 - + + + +
Flag-WT - - + - -
Flag-T188A - - - + -
Flag-S39A - - - - +
43KDa
33KDa
Input: β-actin
(42KDa)
shIGFBP7 - + + + +
Flag-WT - - + - -
Flag-T188A - - - + -
Flag-S39A - - - - +
72KDa
55KDa
Input: GALNT10
(69KDa)

Figure S6F

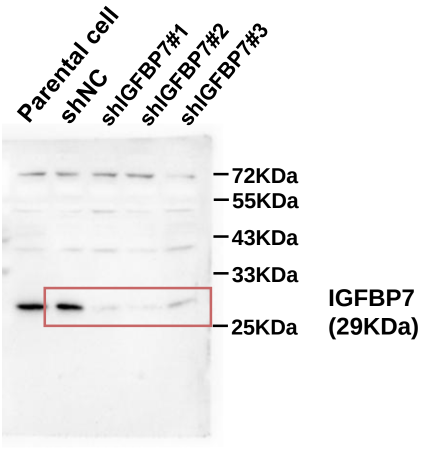
Parental cell
shNC
shIGFBP7#1
shIGFBP7#2
shIGFBP7#3
72KDa
55KDa
43KDa
33KDa
25KDa
IGFBP7
(29KDa)

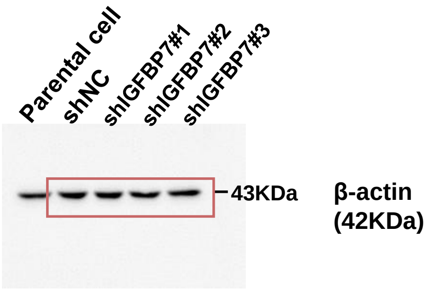
Parental cell
shNC
shIGFBP7#1
shIGFBP7#2
shIGFBP7#3
43KDa
β-actin
(42KDa)

Figure 4C

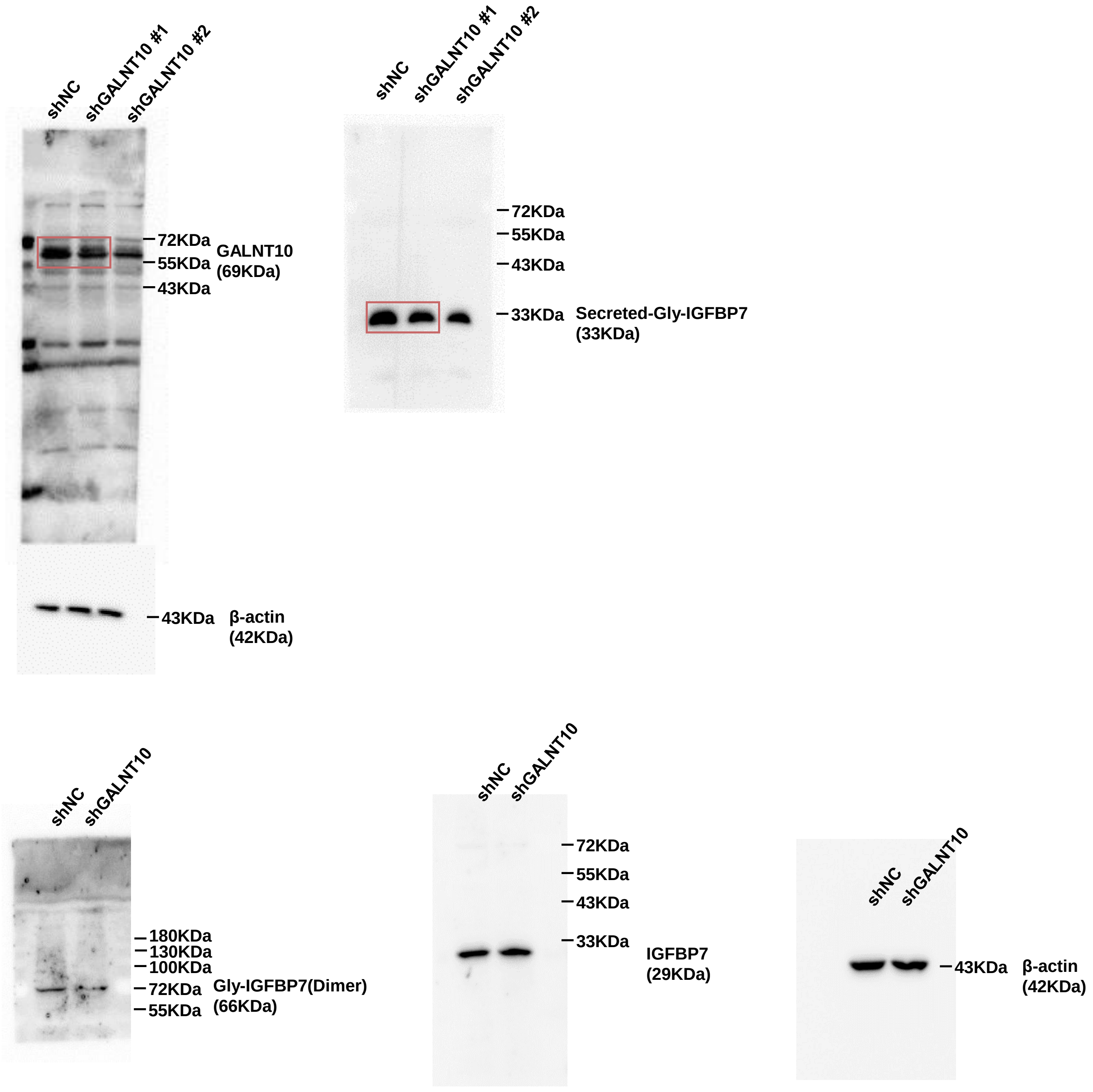
shNC
shGALNT10 #1
shGALNT10 #2
72KDa
55KDa
43KDa
GALNT10
(69KDa)
43KDa
β-actin
(42KDa)
shNC
shGALNT10 #1
shGALNT10 #2
72KDa
55KDa
43KDa
33KDa
Secreted-Gly-IGFBP7
(33KDa)
shNC
shGALNT10
180KDa
130KDa
100KDa
72KDa
55KDa
Gly-IGFBP7(Dimer)
(66KDa)
shNC
shGALNT10
72KDa
55KDa
43KDa
33KDa
IGFBP7
(29KDa)
shNC
shGALNT10
43KDa
β-actin
(42KDa)

Figure 4D

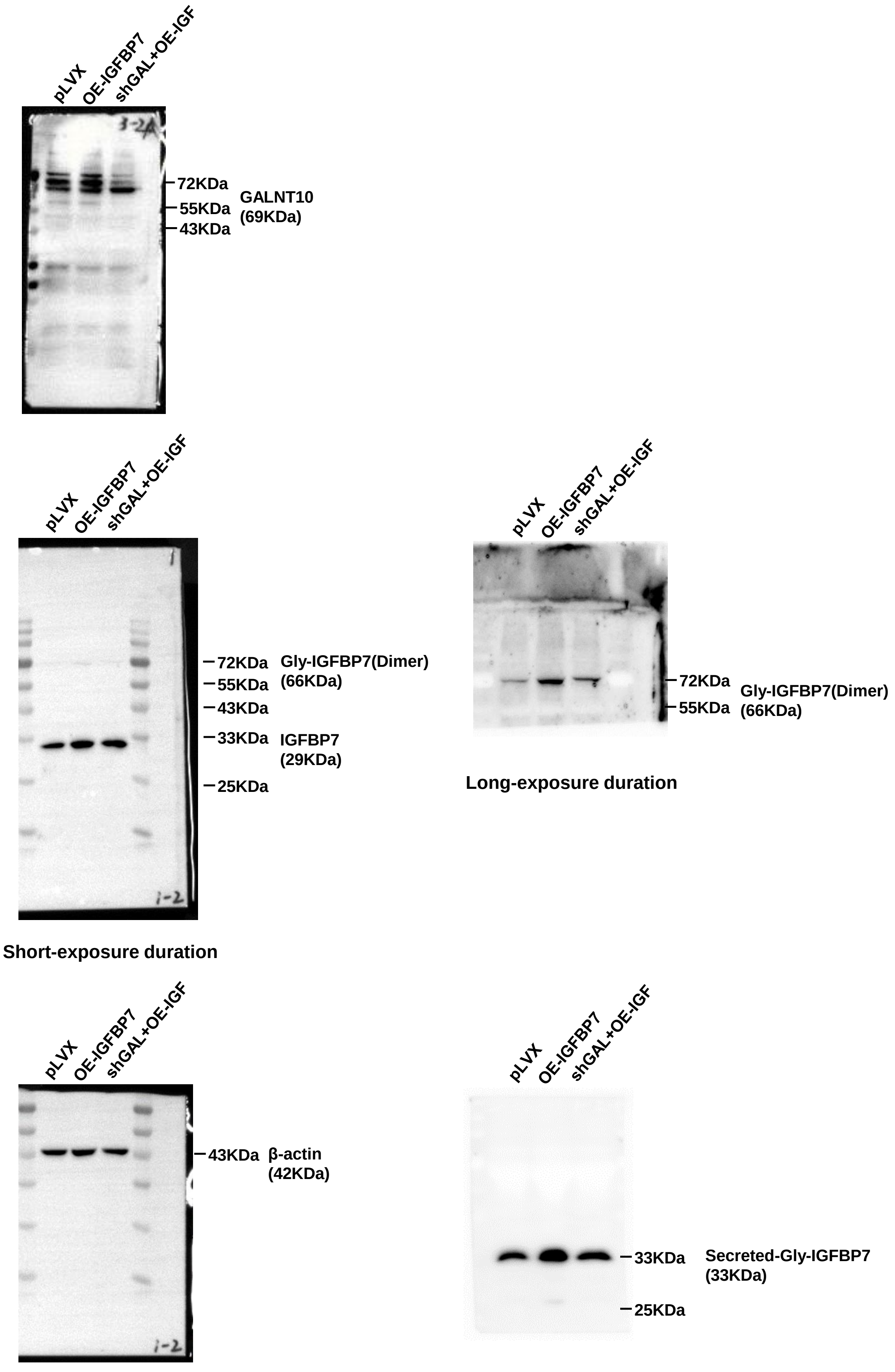
pLVX
OE-IGFBP7
shGAL+OE-IGF
72KDa
55KDa
43KDa
GALNT10
(69KDa)
72KDa
55KDa
43KDa
33KDa
25KDa
Gly-IGFBP7(Dimer)
(66KDa)
IGFBP7
(29KDa)
Short-exposure duration
72KDa
55KDa
Gly-IGFBP7(Dimer)
(66KDa)
Long-exposure duration
43KDa
β-actin
(42KDa)
33KDa
25KDa
Secreted-Gly-IGFBP7
(33KDa)

Figure 4N

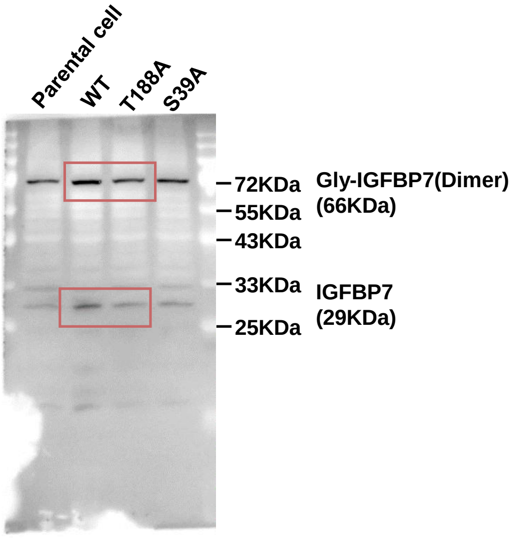
Parental cell
WT
T188A
S39A
72KDa
55KDa
43KDa
33KDa
25KDa
Gly-IGFBP7(Dimer)
(66KDa)
IGFBP7
(29KDa)

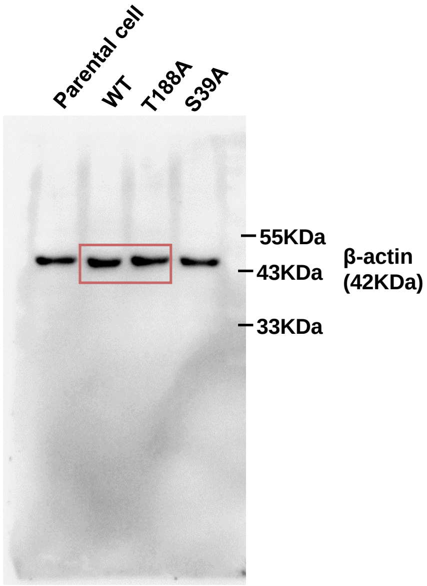
Parental cell
WT
T188A
S39A
55KDa
43KDa
33KDa
β-actin
(42KDa)

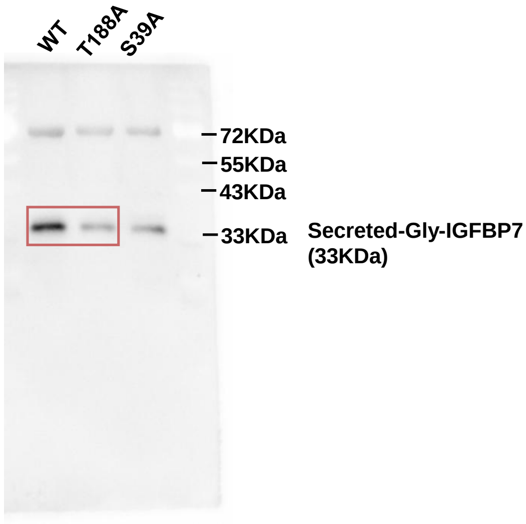
WT
T188A
S39A
72KDa
55KDa
43KDa
33KDa
Secreted-Gly-IGFBP7
(33KDa)

Figure 5A

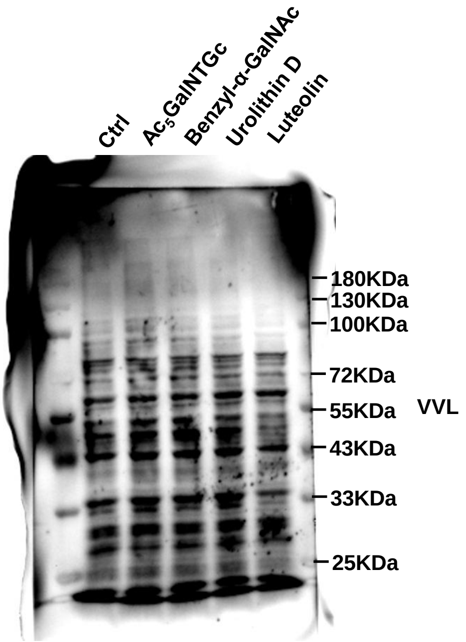
Ctrl
Ac5GalNTGc
Benzyl-α-GalNAc
Urolithin D
Luteolin
180KDa
130KDa
100KDa
72KDa
55KDa
43KDa
33KDa
25KDa
VVL

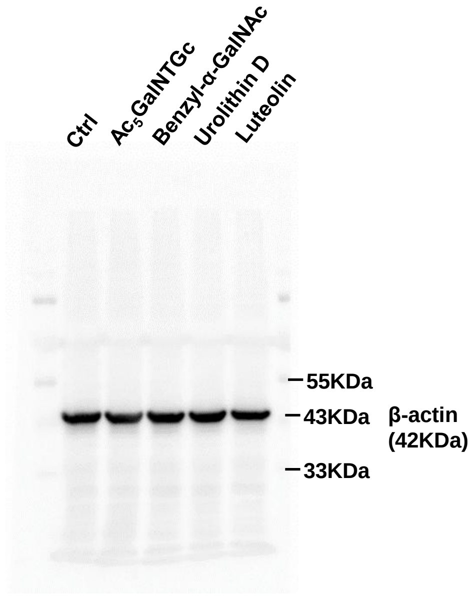
Ctrl
Ac5GalNTGc
Benzyl-α-GalNAc
Urolithin D
Luteolin
55KDa
43KDa
33KDa
β-actin (42KDa)

Figure 5B

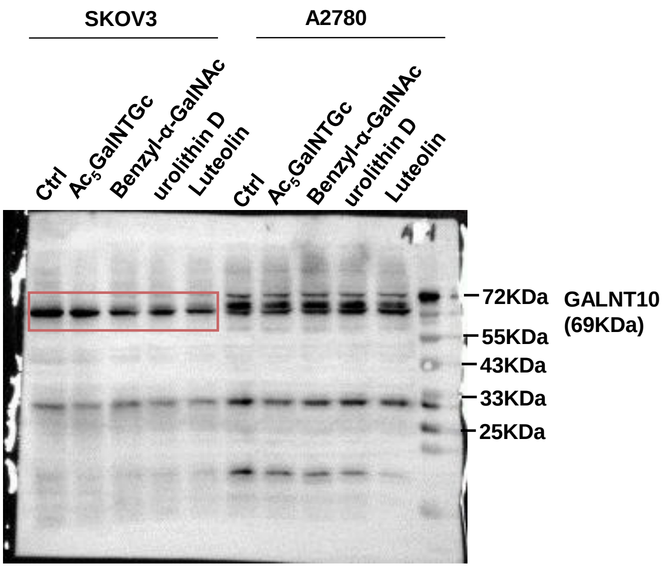

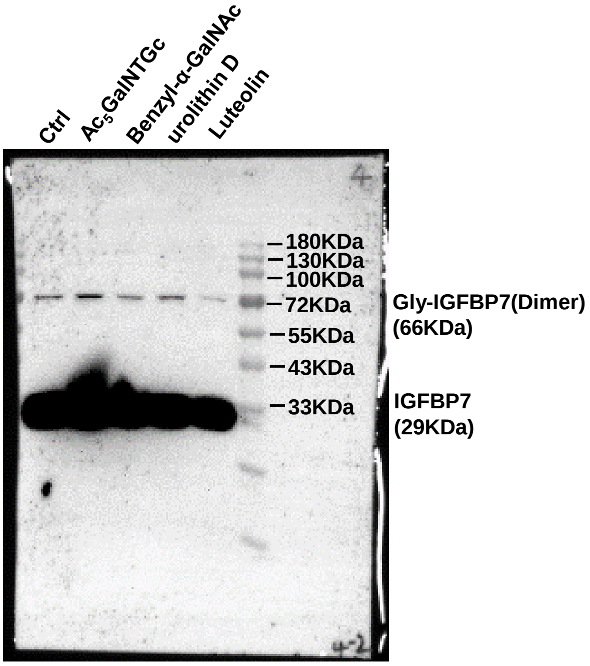

Long-exposure duration

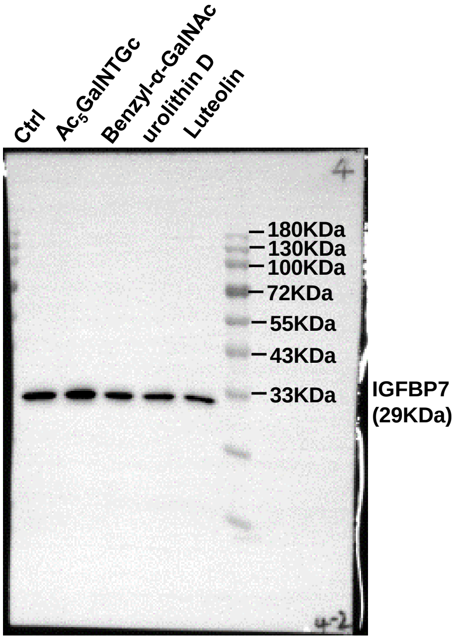

Short-exposure duration

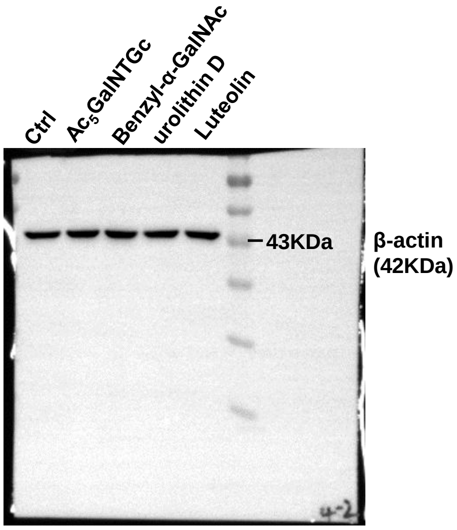

Figure 5E

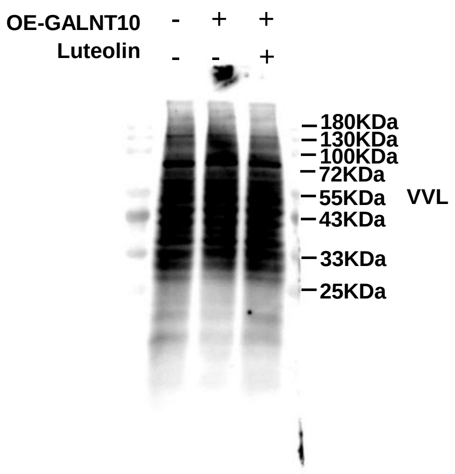
OE-GALNT10 - + +
Luteolin - - +
180KDa
130KDa
100KDa
72KDa
55KDa
43KDa
33KDa
25KDa
VVL

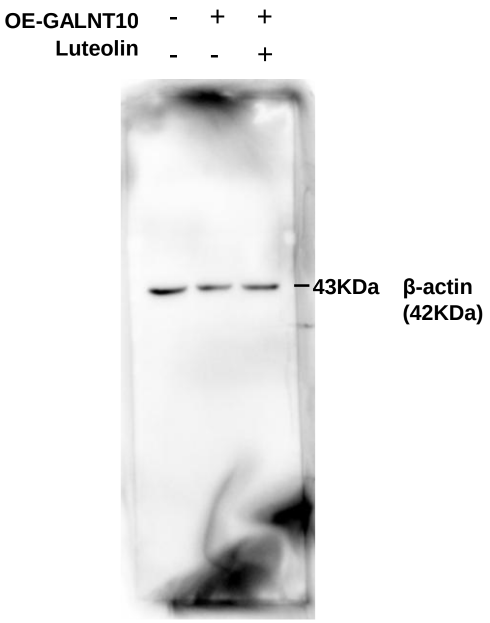
OE-GALNT10 - + +
Luteolin - - +
43KDa
β-actin
(42KDa)

Figure S10B

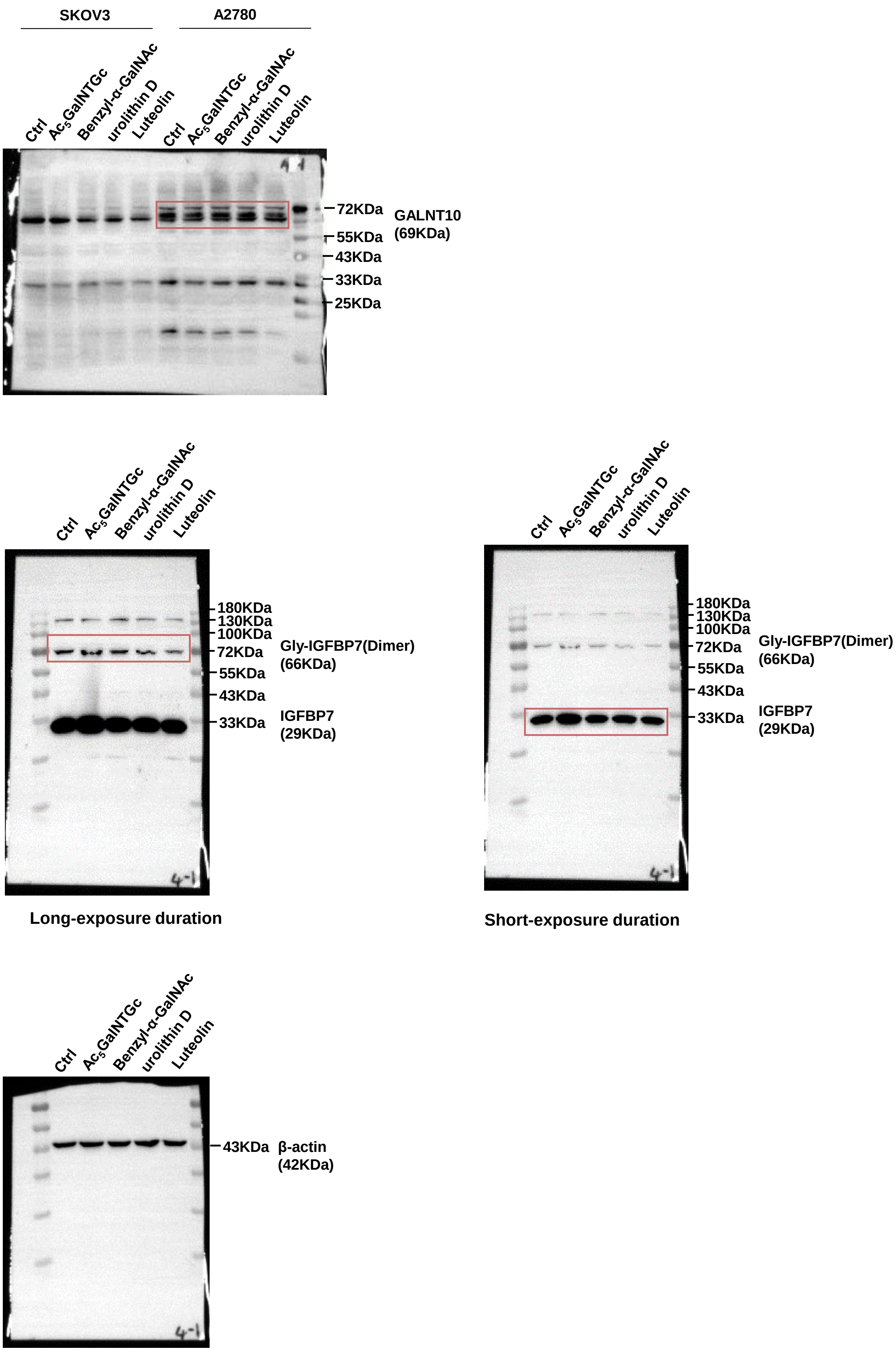
SKOV3
A2780
Ctrl
Ac$_5$GalNTGc
Benzyl-α-GalNAc
urolithin D
Luteolin
Ctrl
Ac$_5$GalNTGc
Benzyl-α-GalNAc
urolithin D
Luteolin
72KDa
55KDa
43KDa
33KDa
25KDa
GALNT10
(69KDa)
Ctrl
Ac$_5$GalNTGc
Benzyl-α-GalNAc
urolithin D
Luteolin
180KDa
130KDa
100KDa
72KDa
55KDa
43KDa
33KDa
Gly-IGFBP7(Dimer)
(66KDa)
IGFBP7
(29KDa)
Long-exposure duration
Ctrl
Ac$_5$GalNTGc
Benzyl-α-GalNAc
urolithin D
Luteolin
180KDa
130KDa
100KDa
72KDa
55KDa
43KDa
33KDa
Gly-IGFBP7(Dimer)
(66KDa)
IGFBP7
(29KDa)
Short-exposure duration
Ctrl
Ac$_5$GalNTGc
Benzyl-α-GalNAc
urolithin D
Luteolin
43KDa
β-actin
(42KDa)
