# Supplementary material for: GALNT10 Affects O‐Glycosylation of IGFBP7 to Promote Tumor Vascular Remodeling and Metastasis of Ovarian Cancer
Source: Adv Sci (Weinh). 2026 Feb 4;13(19):e16106. doi: 10.1002/advs.202516106 (PMC13045431; doi:10.1002/advs.202516106)
Supplement: Supplementary file 3 — Supporting File 3: advs74014‐sup‐0003‐FigureS1‐S13.docx. [file ADVS-13-e16106-s002.docx]

**GALNT10 affects O-glycosylation of IGFBP7 to promote tumor vascular remodeling and metastasis of ovarian cancer**

Yanan Zhang^1,2,3#^, Ayala Zuha^1,2#^, Zhangxin Wu^1,2#^, Aiping Luo^4^, Bixia Jin^1,2^, Qinkun Sun^1,2^, Yuan Li^1,2^, Qiyu Liu^1,2*^, Hongyan Guo^1,2*^, Chunliang Shang^1,2*^

**Affiliations:**

^1^ Department of Obstetrics and Gynecology, Peking University Third Hospital, Beijing 100191, China.

^2^ National Clinical Research Center for Obstetrics and Gynecology, Beijing, 100191, China.

^3^ State Key Laboratory of Vascular Homeostasis and Remodeling, Peking University Third Hospital, Beijing 100191, China.

^4^ State Key Lab of Molecular Oncology, National Cancer Center/National Clinical Research Center for Cancer/Cancer Hospital, Chinese Academy of Medical Sciences and Peking Union Medical College, Beijing 100021, China.

^#^ These authors contributed equally to this work and all should be considered co-first authors.

Corresponding author：

Chunliang Shang, shangchl@bjmu.edu.cn, Gynecological Ward, No. 49, Garden North Road, Haidian District, Beijing, China, 100191.

Hongyan Guo, bysyghy@163.com, Gynecological Ward, No. 49, Garden North Road, Haidian District, Beijing, China, 100191.

Qiyu Liu, liuqiyu2015@163.com, Gynecological Ward, No. 49, Garden North Road, Haidian District, Beijing, China, 100191.

The authors declare no potential conflicts of interest.


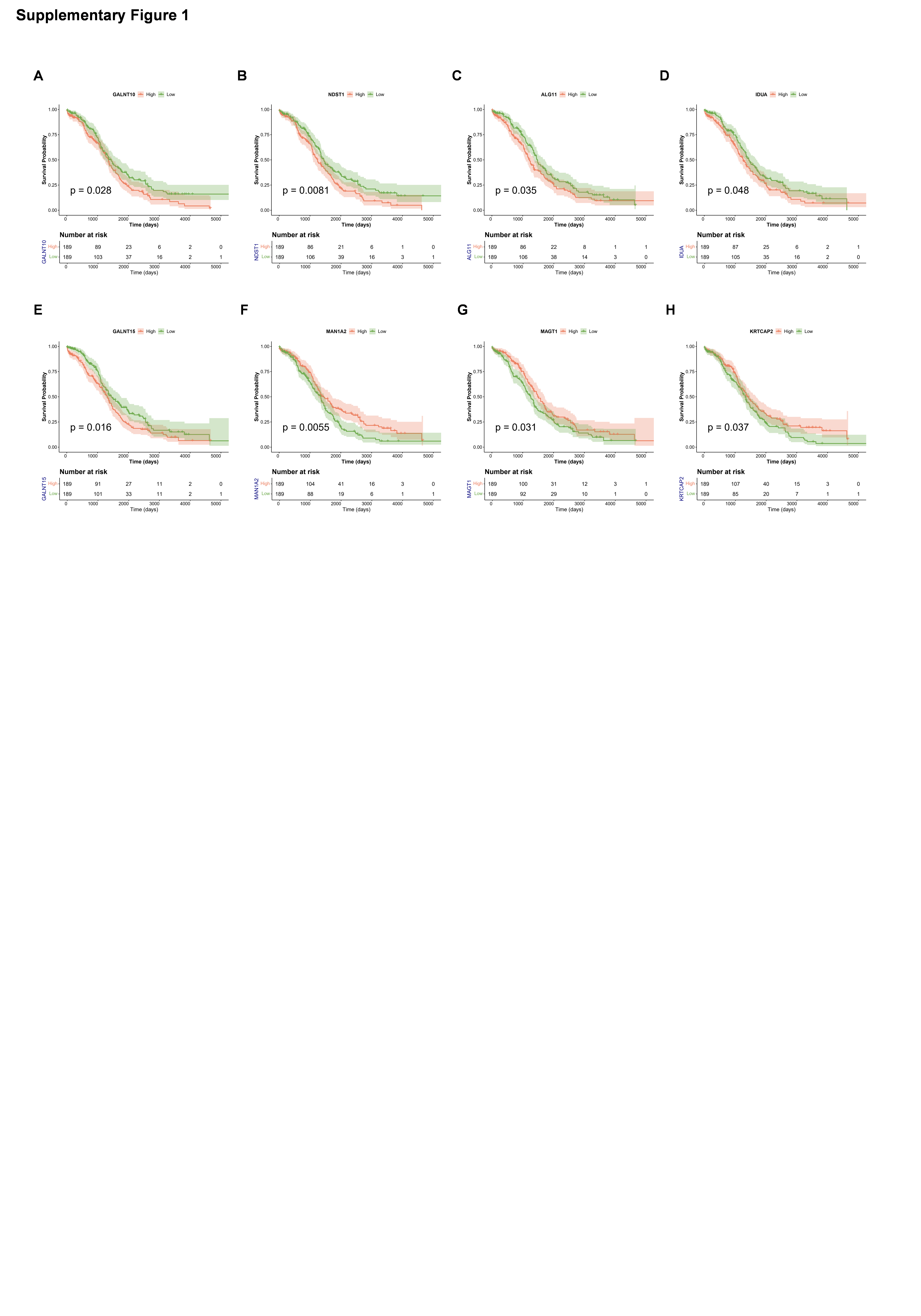


**Figure S1.** (A-H) Survival analysis of 8 genes related to glycan biosynthesis and metabolismg significantly associated with OS in TCGA-OV dataset using Kaplan Meier analysis.


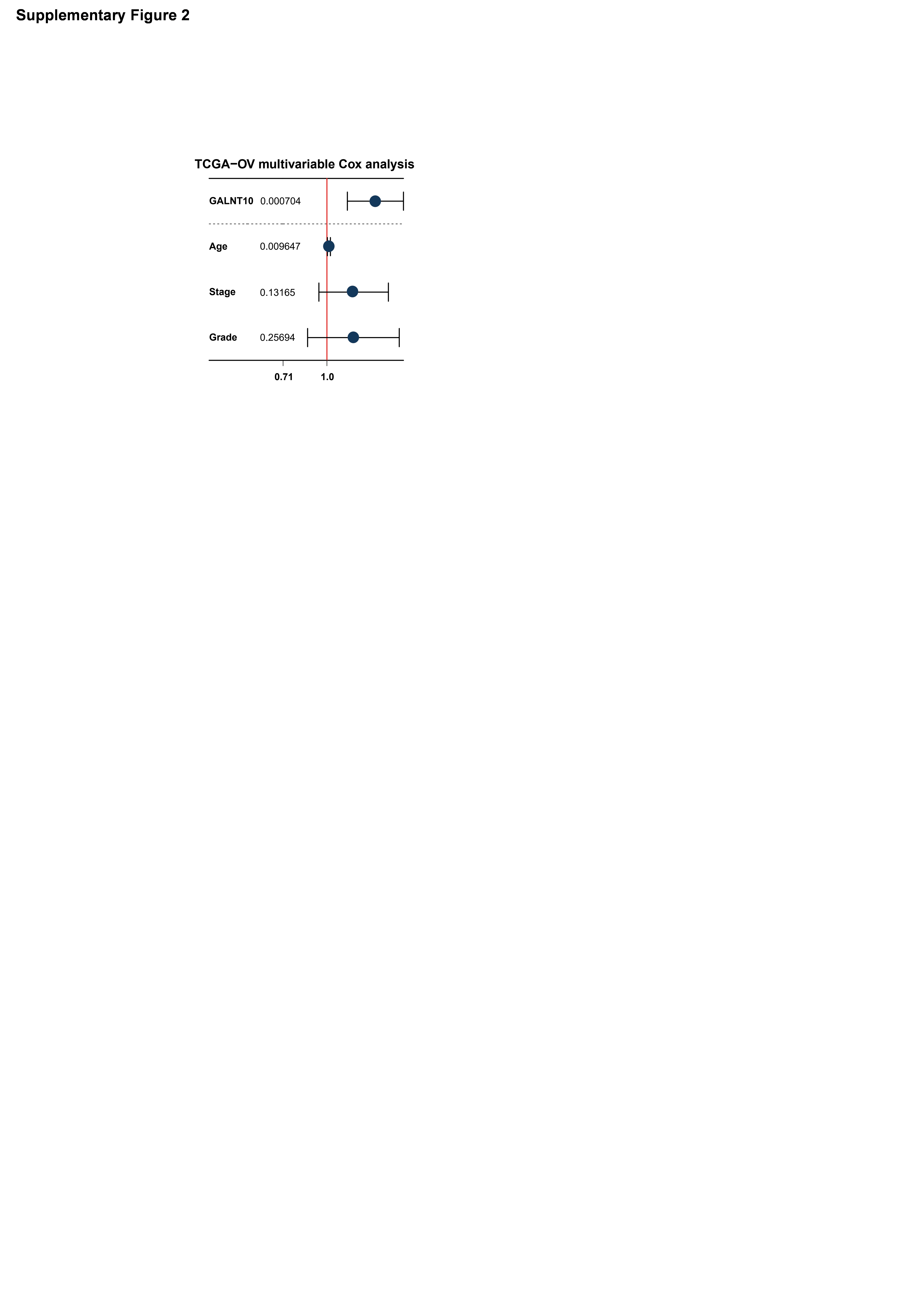


**Figure S2.** Multivariate Cox analysis of TCGA-OV dataset including GALNT10 and several clinical and pathological characteristics.


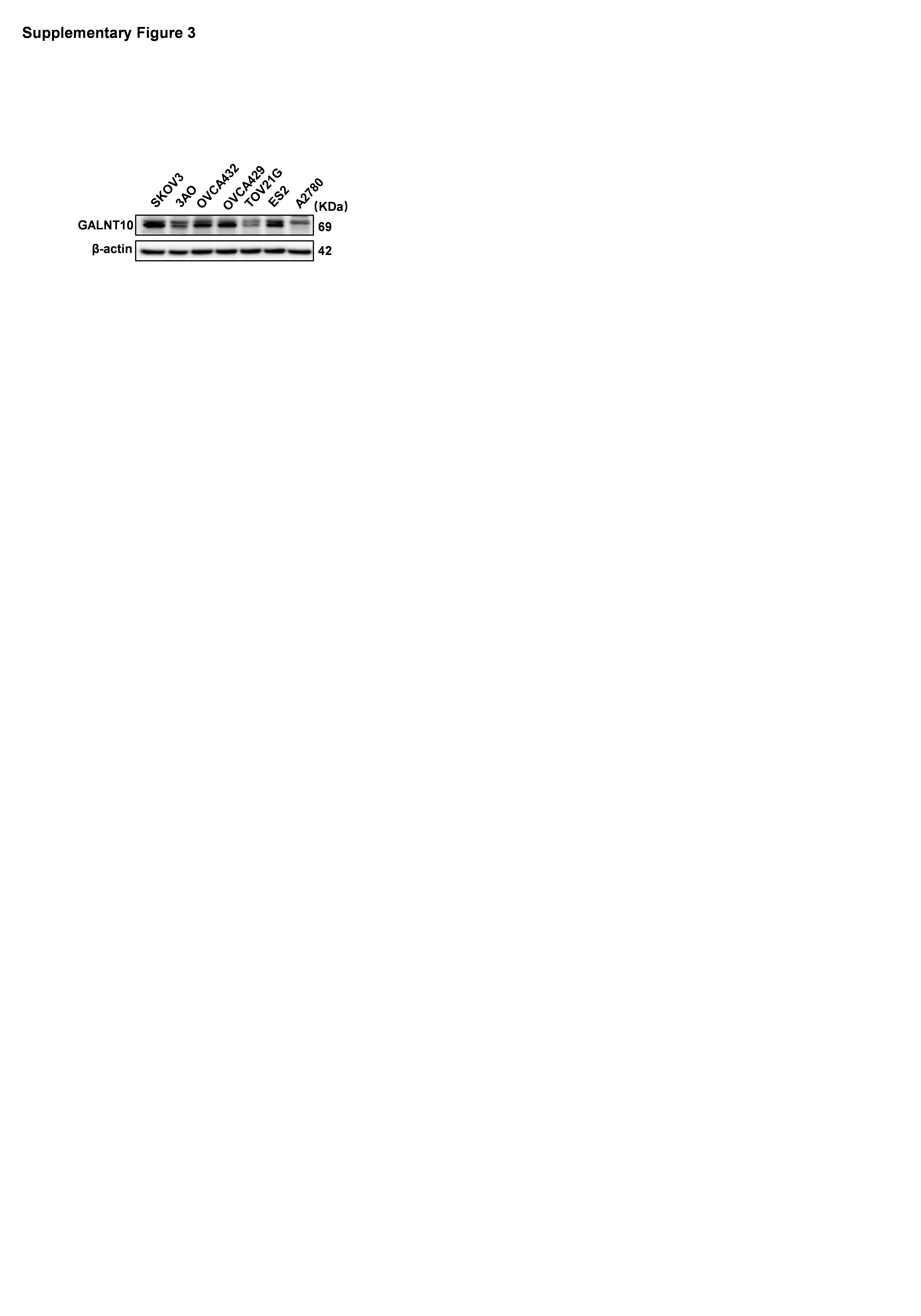


**Figure S3.** GALNT10 expression in 7 ovarian cancer cell lines was detected by Western blot.


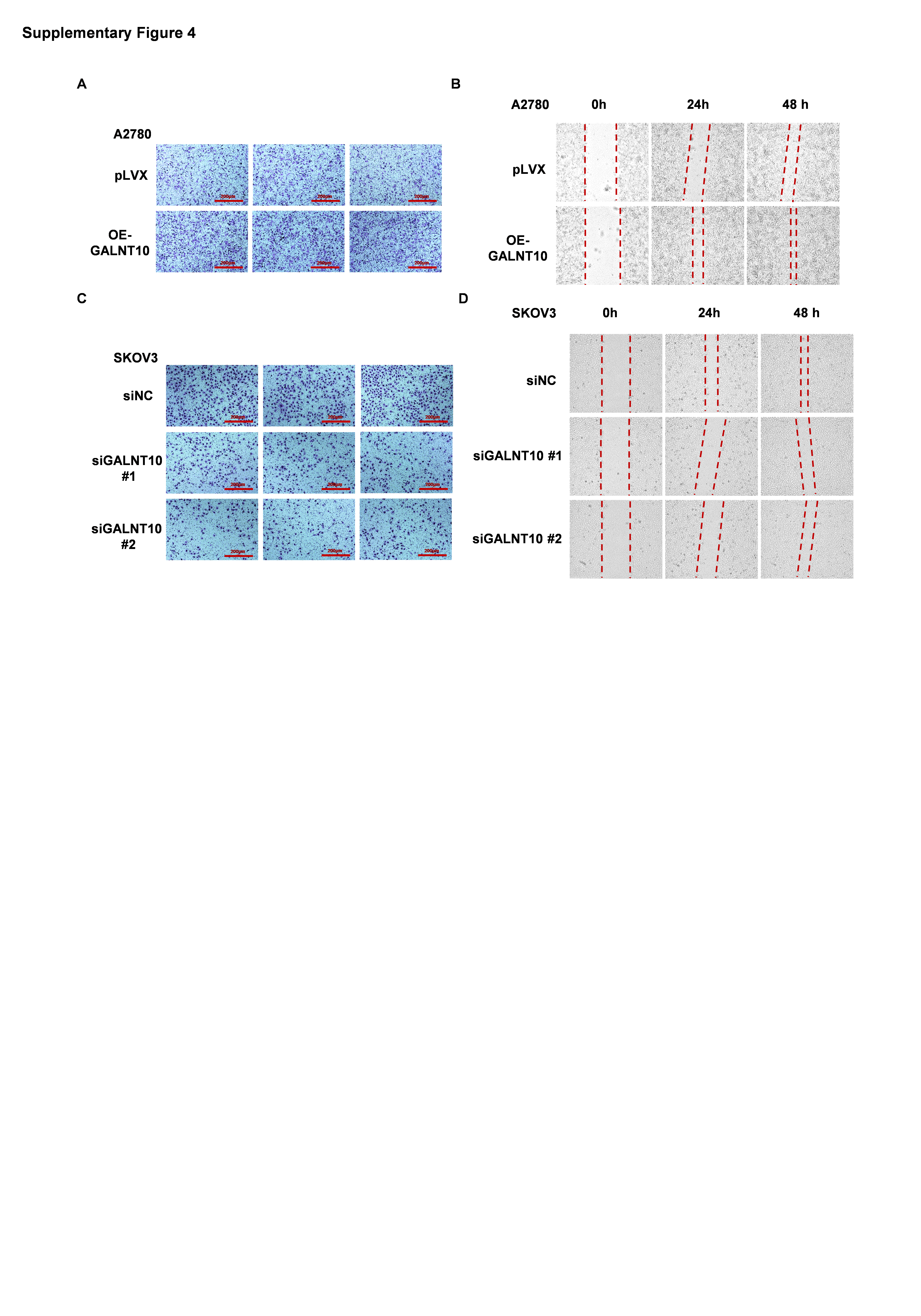


**Figure S4.** (A, B) The Transwell and wound healing images of OE-GALNT10 and control pLVX A2780 cells. (C, D) The Transwell and wound healing images of siGALNT10#1, siGALNT10#2, control siNC SKOV3 cells. The scale shown in the picture is 200μm.


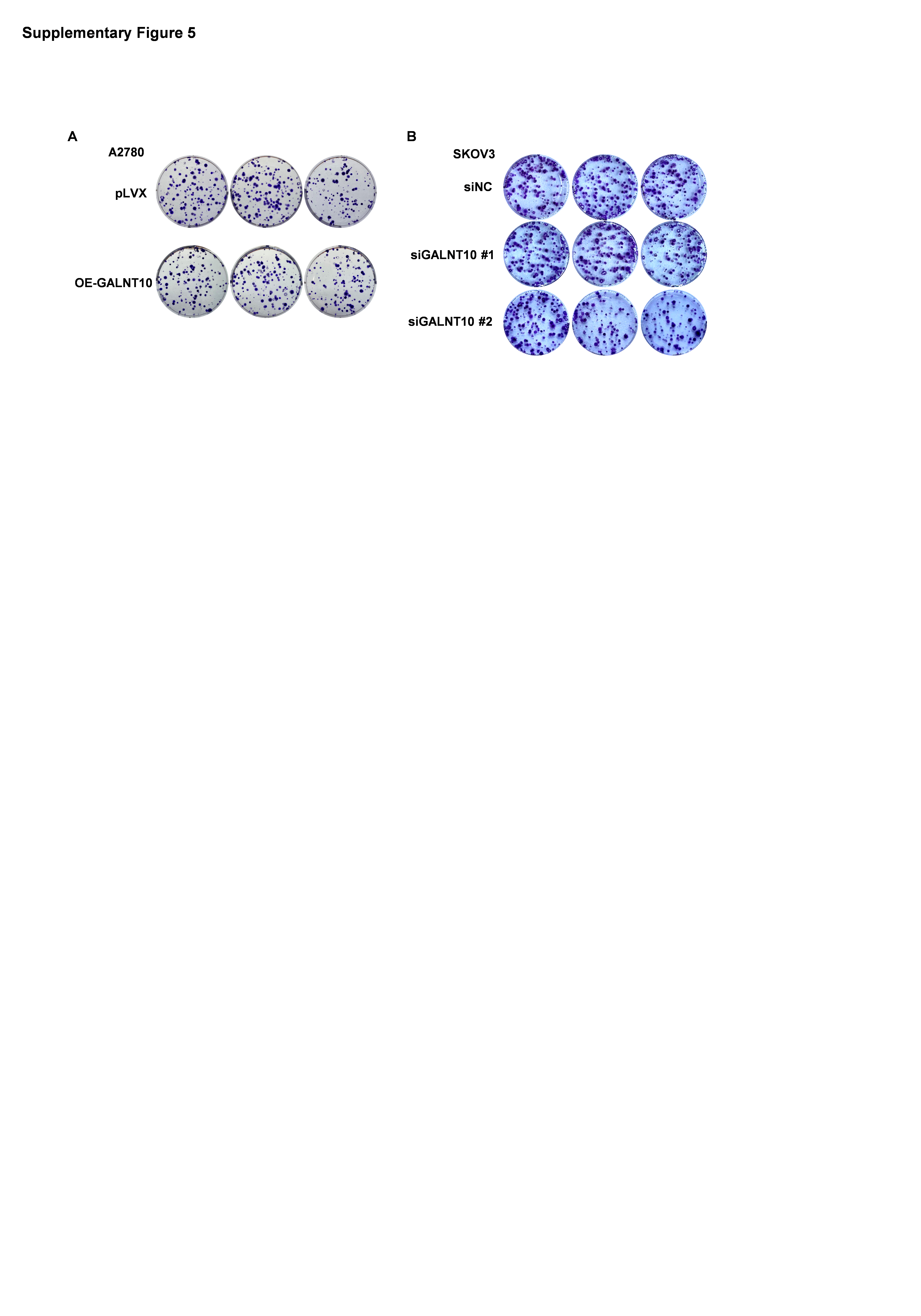


**Figure S5.** (A) The proliferation of OE-GALNT10 and control pLVX A2780 cells were analyzed by clone formation. (B) The proliferation of siGALNT10#1, siGALNT10#2, control siNC SKOV3 cells were analyzed by clone formation.


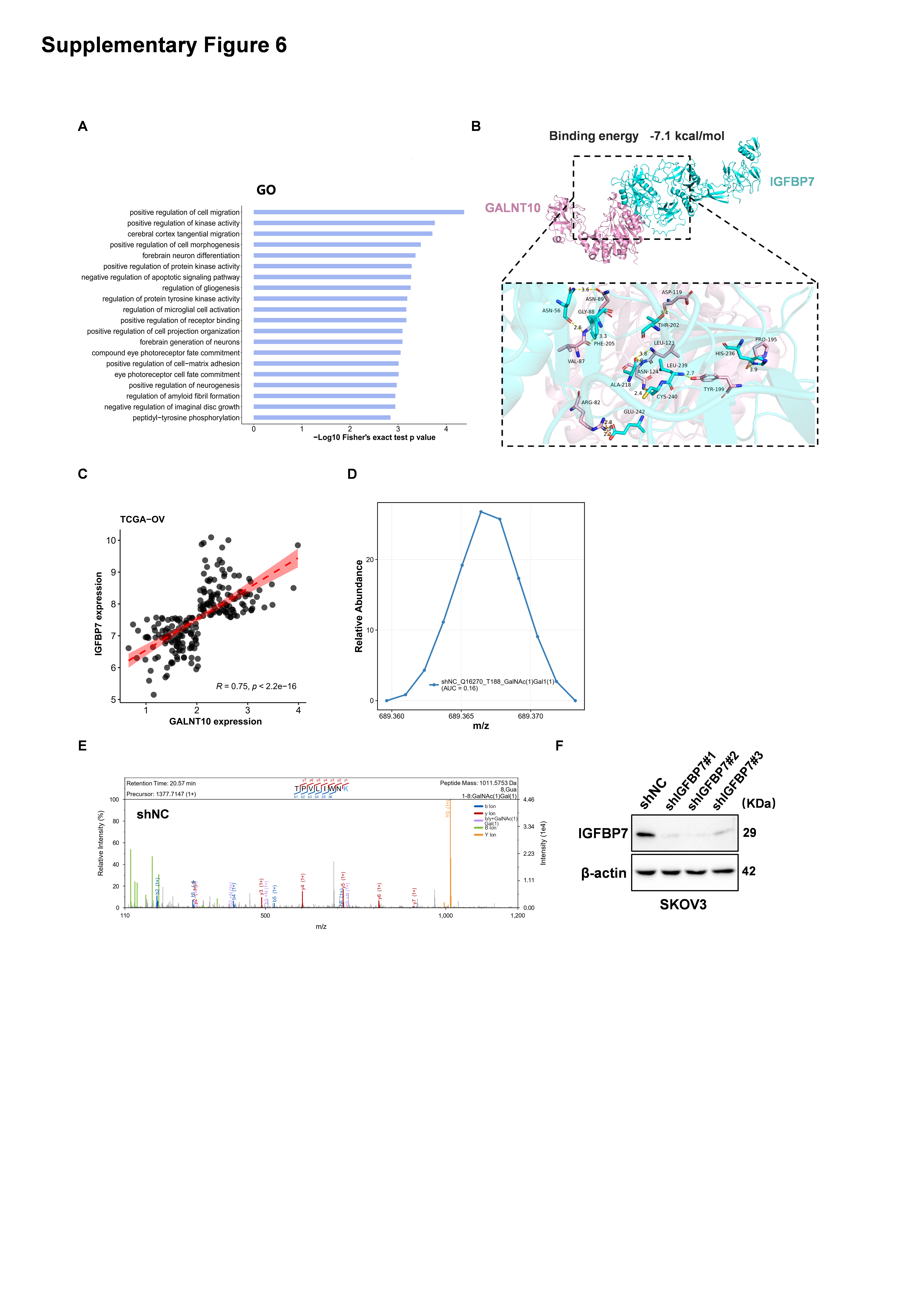


**Figure S6.** (A) GO pathway enrichment of 46 proteins with decreased O-GalNAc glycosylation levels in shGALNT10 SKOV3 cells. (B) The molecular docking analysis of GALNT10 and IGFBP7. (C) The correlation analysis of GALNT10 and IGFBP7 in TCGA-OV dataset. (D) The primary quantitive extracted ion chromatograms (XICs) of the GalNAc(1) Gal(1) glycopeptide at the T188 site of IGFBP7 in shNC and shGALNT10 SKOV3 cells. (E) The GalNAc(1)Gal(1) glycan structures on glycoprotein IGFBP7 at T188 in shNC and shGALNT10 SKOV3 cells characterized by glycoproteomics. (F) IGFBP7 expression in SKOV3 cells transfected with IGFBP7 shRNA lentivirus (shIGFBP7#1-3) or negative control shRNA (shNC) was detected by Western blot.


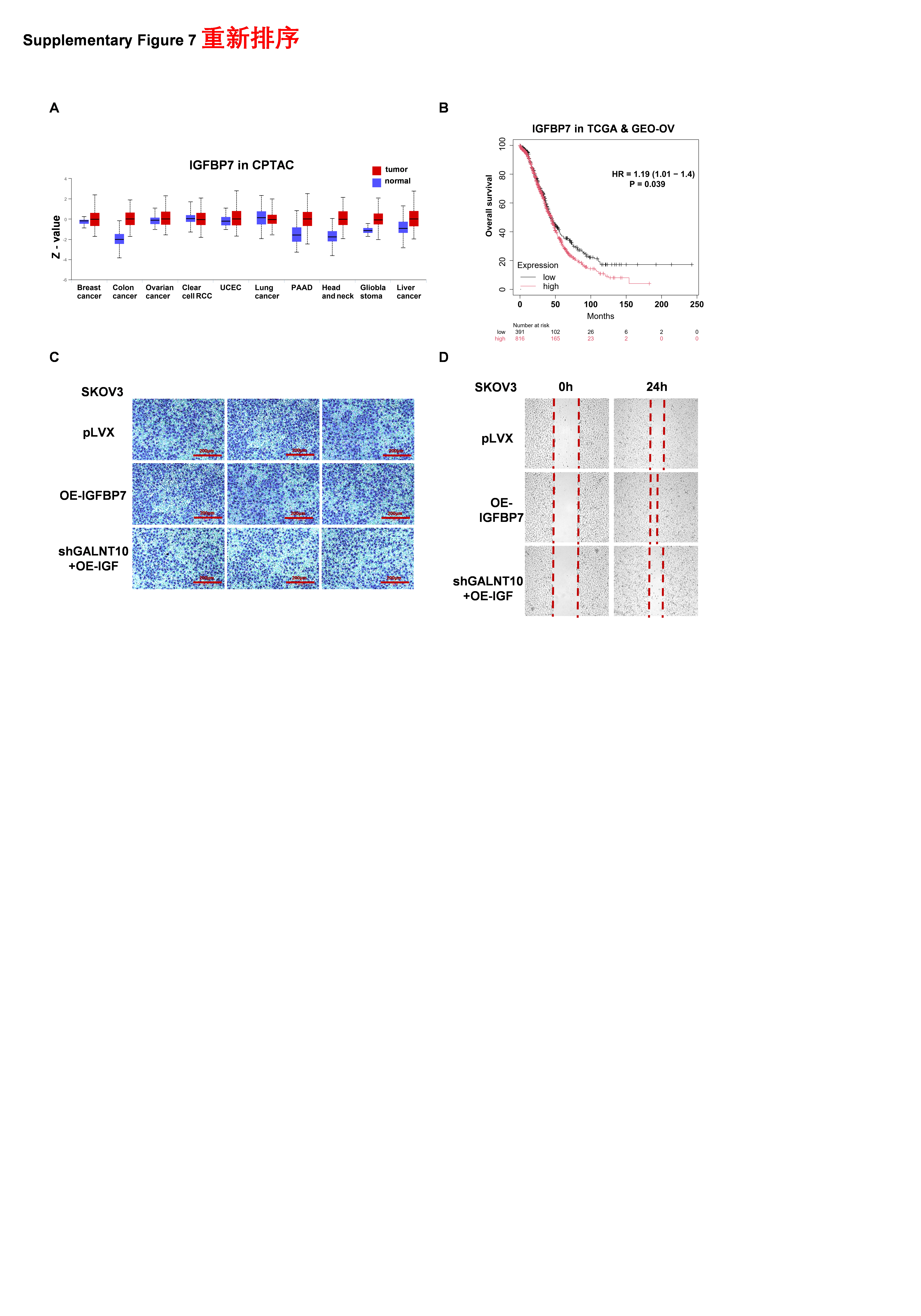


**Figure S7.** (A) IGFBP7 level of pan-cancer and normal tissues in CPTAC. (B) Survival analysis of IGFBP7expression in TCGA and GEO ovarian cancer dataset using the Kaplan-Miere method. (C, D) The original chart of Figure 4E and F. The Transwell (C) and wound healing (D) images of OE-IGFBP7 SKOV3 cells, shGALNT10+OE-IGFBP7 SKOV3 cells and control SKOV3 cells. The scale shown in the picture is 200μm.


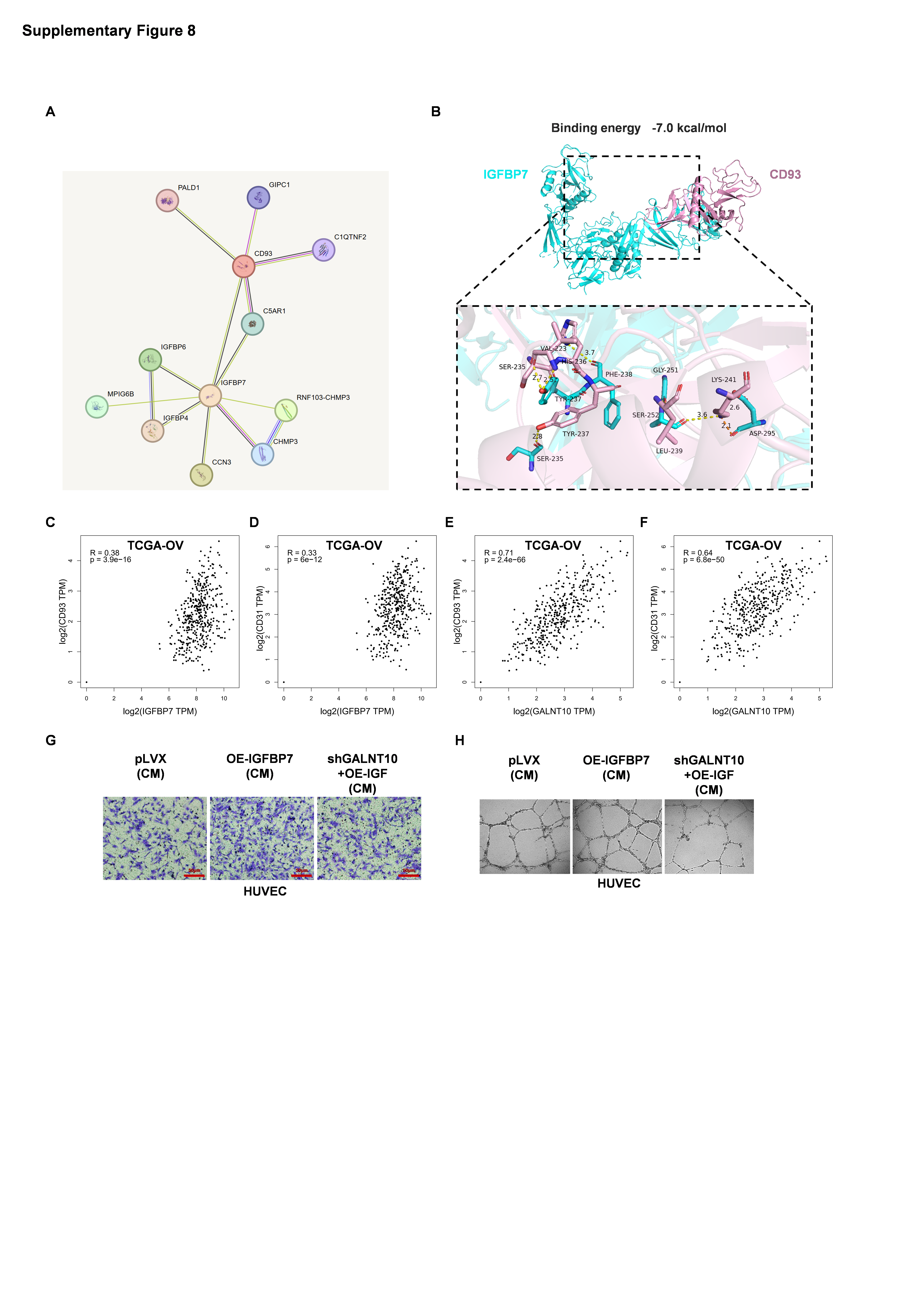


**Figure S8.** (A) The PPI analysis of IGFBP7 and CD93. (B) The molecular docking analysis of IGFBP7 and CD93. (C, D) Correlation analysis of IGFBP7 with CD93 and CD31 in TCGA-OV dataset. (E, F) Correlation analysis of GALNT10 with CD93 and CD31 in TCGA-OV dataset. (G) The original chart of Figure 4G. The invasion abilities of HUVECs incubated with CM of OE-IGFBP7 SKOV3 cells, shGALNT10+OE-IGFBP7 SKOV3 cells, and control SKOV3 cells for 24 hours were analyzed by Transwell. The scale shown in the picture is 50μm. (H) The original chart of Figure 4H and I. The angiogenesis ability of HUVECs incubated with CM of OE-IGFBP7 SKOV3 cells, shGALNT10+OE-IGFBP7 SKOV3 cells, and control SKOV3 cells for 24 hours was analyzed by Tube formation.


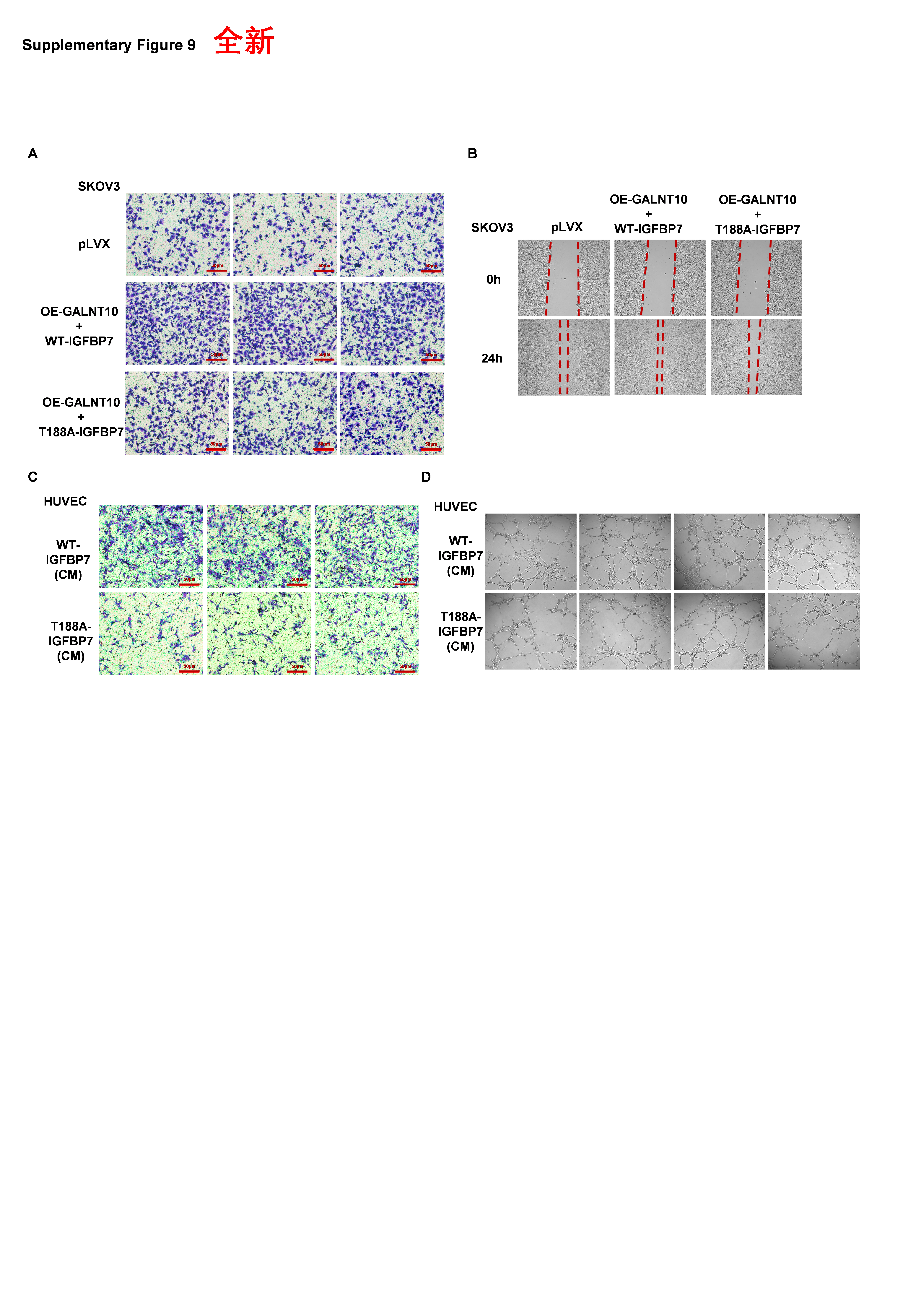


**Figure S9.** (A, B) All the original chart of Figure 4N and O. (C) All the original chart of Figure 4P. (D) All the original chart of Figure 4Q and R. The scale shown in the picture is 50μm.


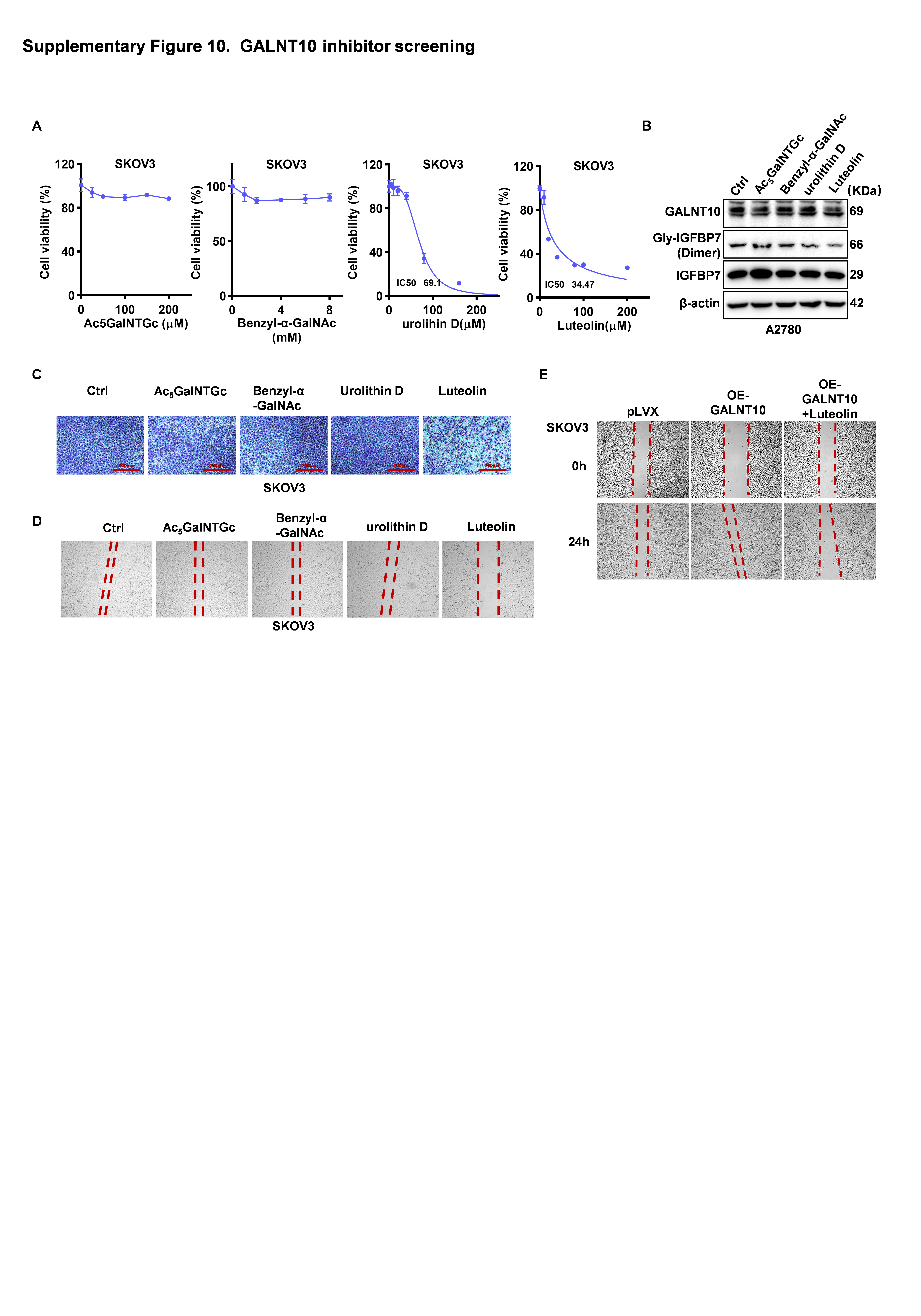


**Figure S10.** (A) The viability of SKOV3 cells treated with the indicated concentrations of GALNTs inhibitors evaluated by CCK8. (B) The glycoslyted IGFBP7 level inhibition of GALNTs inhibitors in A2780 cells was detected by Western Blot. (C, D) The Transwell (C) and wound healing (D) images of SKOV3 cells treated with 10 μM GALNTs inhibitors. The scale shown in the picture is 200μm. (E) The original chart of Figure 5F.


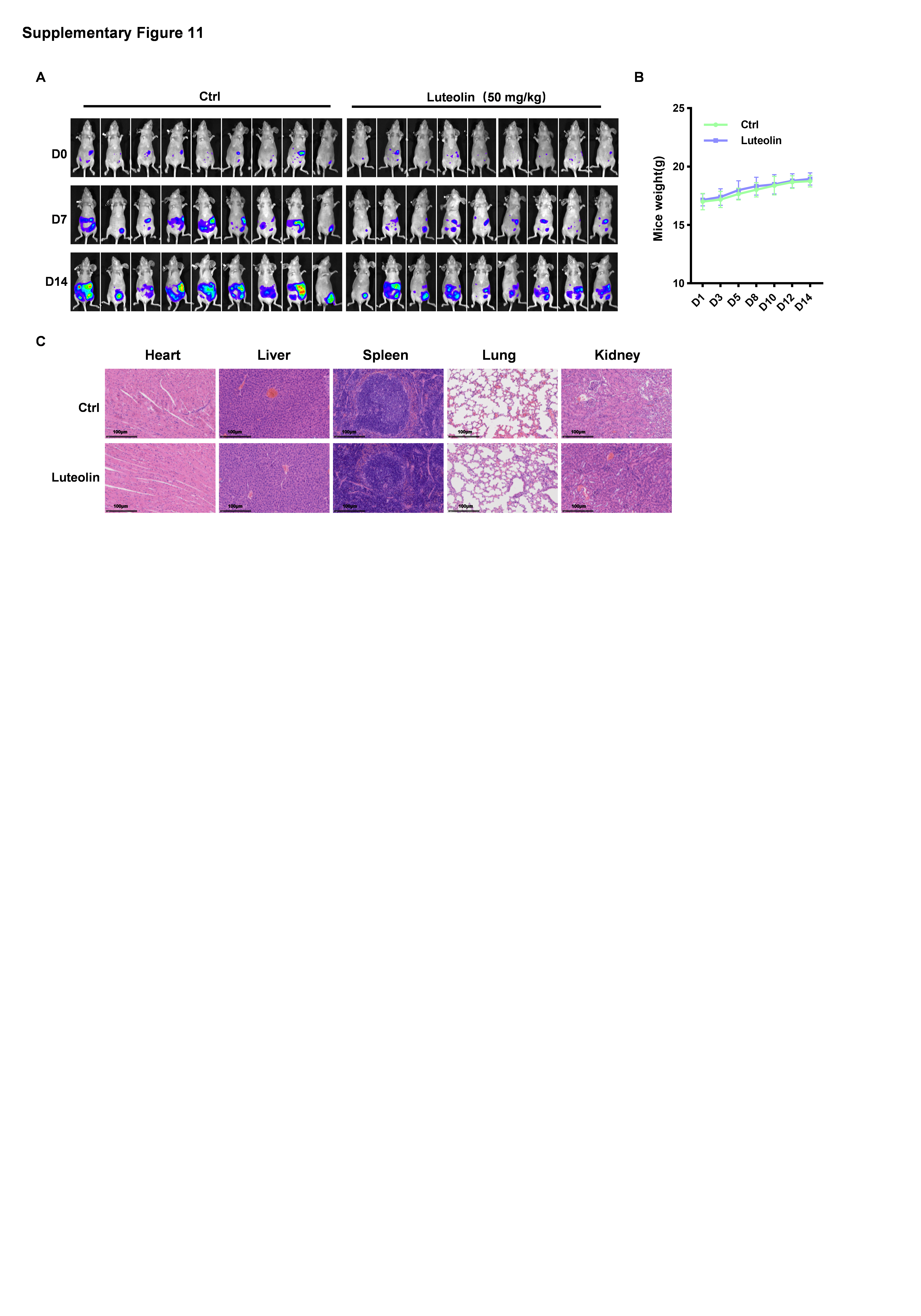


**Figure S11.** (A) Bioluminescence images of mice bearing abdominal SKOV3-derived tumors after treatment as indicated (n = 9 mice per group). (B) The mice weight in Luteolin treated group and control group (n = 9 mice per group). (C) Representative HE staining images of these major organs in Luteolin treated group and control group. The scale shown in the picture is 250μm.


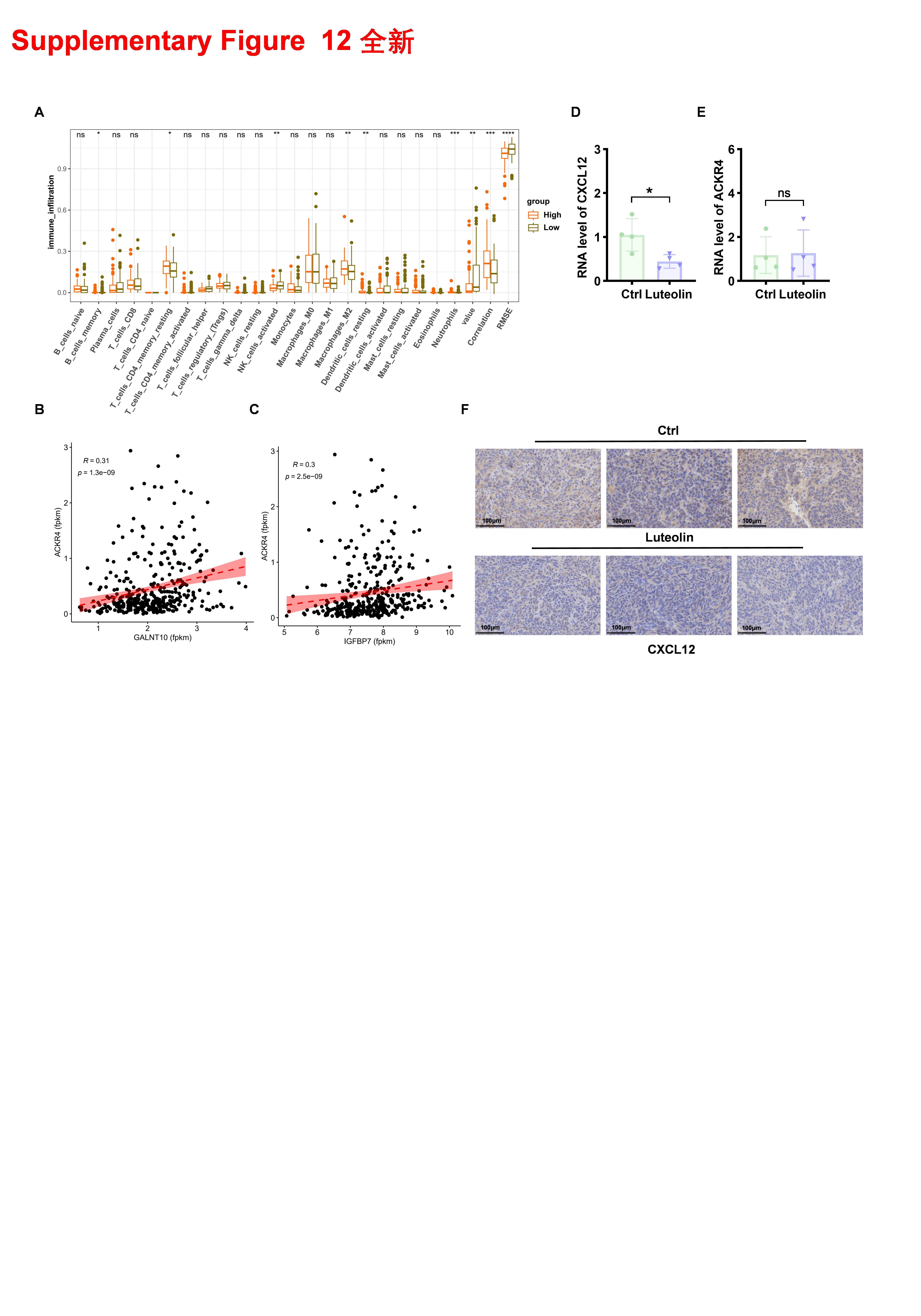


**Figure S12.** (A) The proportions of immune cell types across GALNT10^high^ and GALNT10^low^ groups in the TCGA-OV dataset using CIBERSORT. (B, C) The correlation analysis of IGFBP7, GALNT10 and ACKR4 in TCGA-OV dataset. (D, E) The RNA level of CXCL12 and ACKR4 in orthotopic tumor of Luteolin group and negstive control group were detected by qPCR. (H) CXCL12 expression of orthotopic tumors were detected by IHC. The scale shown in the picture is 100μm. Statistical analysis was performed by a two-tailed, unpaired Student’s t-test. *P<0.05, ns means P>0.05.


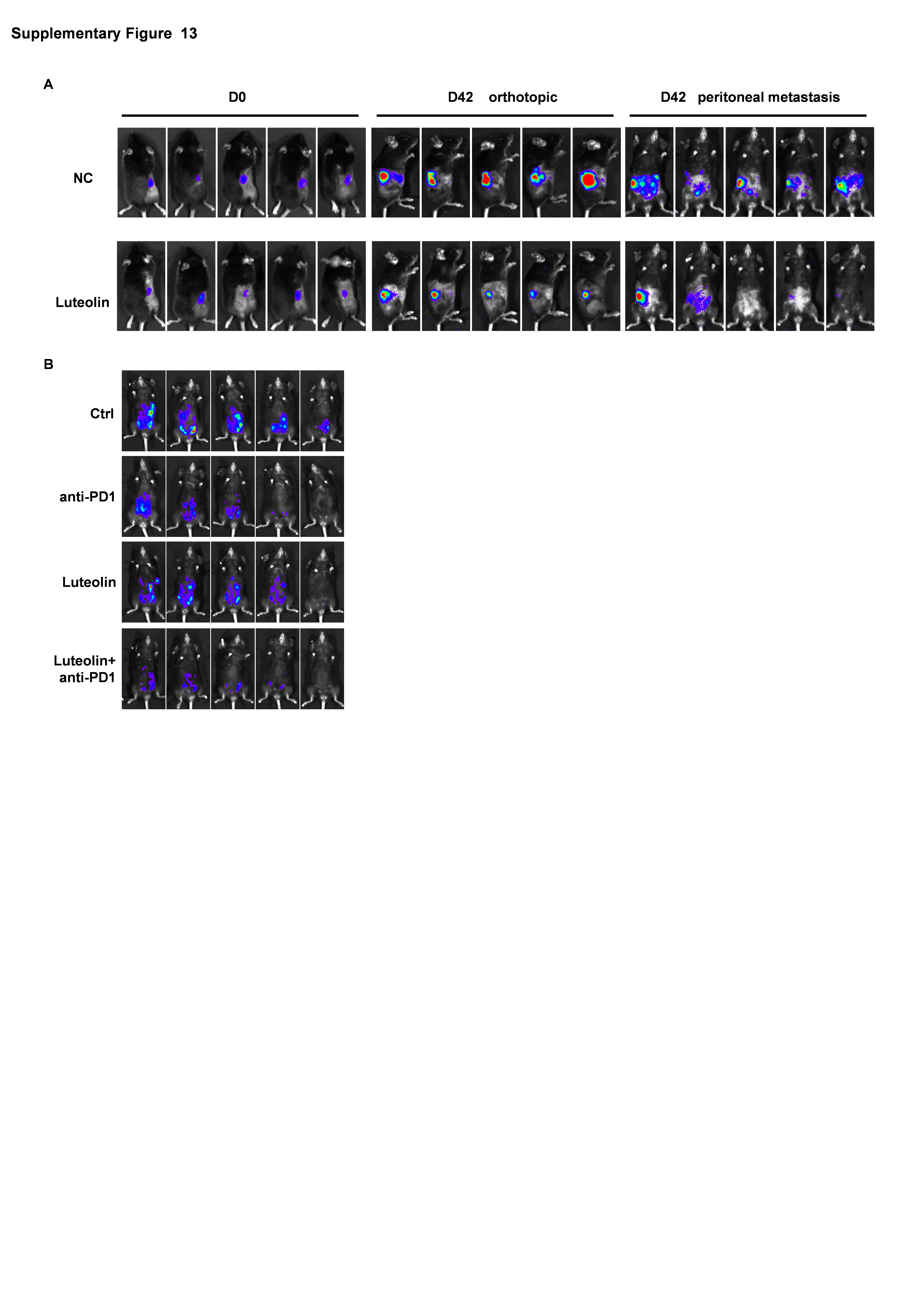


**Figure S13.** (A) Complete information display of Figure 6G, the bioluminescence images of mice bearing orthotopic ID8-derived tumors after treatment as indicated (n = 5 mice per group). (B) Complete information display of Figure 6N, the bioluminescence images of mice bearing peritoneal metastasis ID8-derived tumors after treatment as indicated (n = 5 mice per group).
